# Supplementary material for: COVID-19 and crime: Analysis of crime dynamics amidst social distancing protocols
Source: PLoS One. 2021 Apr 1;16(4):e0249414. doi: 10.1371/journal.pone.0249414 (PMC8016314; doi:10.1371/journal.pone.0249414)
Supplement: S1 File — (PDF) [file pone.0249414.s001.pdf]

COVID-19 and crime: Analysis of crime dynamics amidst social distancing protocols

Shelby M. Scott*[1¶#a], Louis J. Gross[1,2&]

1 Department of Ecology and Evolutionary Biology, University of Tennessee, Knoxville, Knoxville, Tennessee, United States of America
2 National Institute for Mathematical and Biological Synthesis, Knoxville, Tennessee, United States of America

¶SMS contributed Conceptualization, Methodology, Formal Analysis, Data Curation, Writing - Original Draft, Validation Visualization
& LJG contributed Conceptualization, Methodology, Resources, Writing - Review & Editing, Supervision, Funding acquisition
#a Current Address: Department of Ecology and Evolutionary Biology, University of Tennessee, Knoxville, 569 Dabney Hall, Knoxville, TN 37996, USA
* sscott41@vols.utk.edu (SMS)

Supporting information

All data and code files used in this analysis are available through GitHub at `https://github.com/shelbymscott/COVIDandCrime`.

Further Background Information

Social Distancing Adherence The purpose of this study was to determine how changes in social dynamics in response to stay at home orders altered the crime levels in various cities. To test the impact of behavioral changes, it is helpful to know when those behavioral changes were most stark. We chose to analyze the two weeks following implementation of stay at home orders because the minimum value for all three metrics and across all three cities provided by Unacast occurred within the short period of time following the stay-at-home order (Figs S1-S3).

Demographics of Chicago, Baltimore, and Baton Rouge Chicago, Baltimore, and Baton Rouge are cities of differing size, race makeups, and socio-economic characteristics. All three cities are often cited for their crime levels and they are all above the national average in terms of poverty. Below are details regarding demographic information, taken from the Census Bureau.

City	Population Size	% White	% Black	% American Indian or Alaskan	% Asian	% 2+ Races	% Hispanic/ Latino	% White Non-Hispanic/Latino	% In Poverty	Land mass (mi^2)	Population Density
Chicago	2,693,976	49.4	30.1	0.3	6.4	2.7	29.0	32.8	19.5	227.36	11,841.8
Baltimore	593,490	31.8	62.7	0.5	2.7	2.2	5.7	27.7	18.9	80.94	7,671.5
Baton Rouge	220,236	38.7	55.0	0.2	3.2	1.4	3.7	36.6	25.2	76.95	2,982.5

Table S1. Demographics of Chicago, Baltimore, and Baton Rouge. Chicago is the largest of the three cities, followed by Baltimore and Baton Rouge. The same pattern holds for population density, but there are similarities in racial breakdowns and poverty levels across the three cities.

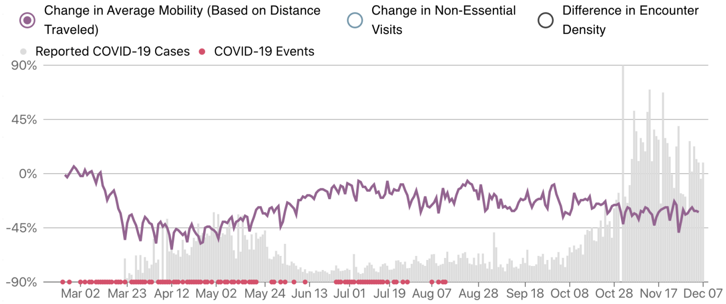

(a) Average Mobility

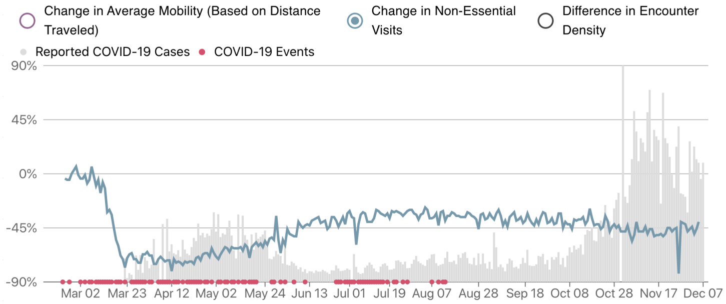

(b) Non-Essential Visits

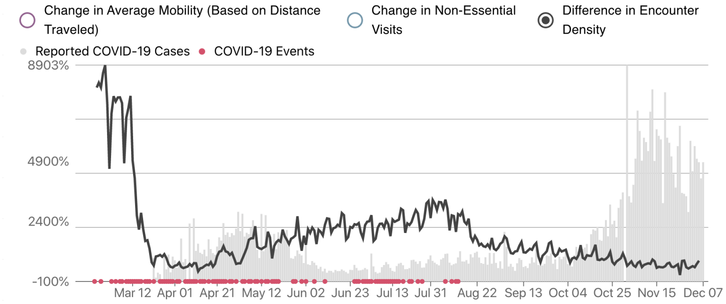

(c) Encounter Density

Fig S1. Metrics for adherence to social distancing guidelines in Chicago, as provided by Unacast's dashboard. The most extreme drops in all three metrics occurred after the stay at home order was implemented.

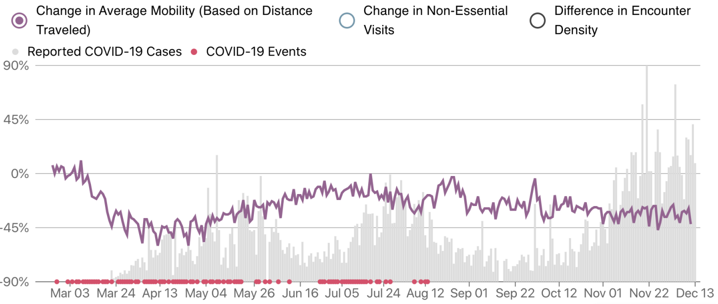

(a) Average Mobility

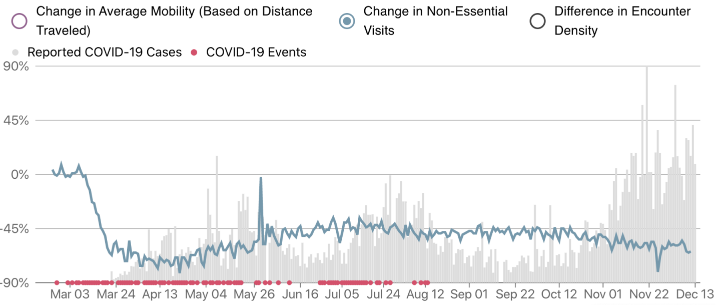

(b) Non-Essential Visits

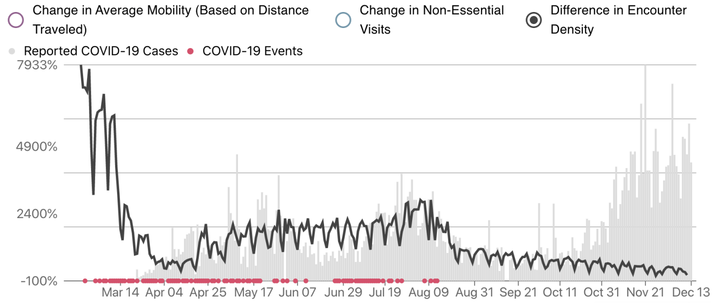

(c) Encounter Density

Fig S2. Metrics for adherence to social distancing guidelines in Baltimore, as provided by Unacast's dashboard. The most extreme drops in all three metrics occurred after the stay at home order was implemented.

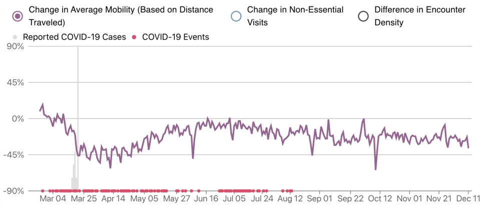

(a) East Baton Rouge Average Mobility

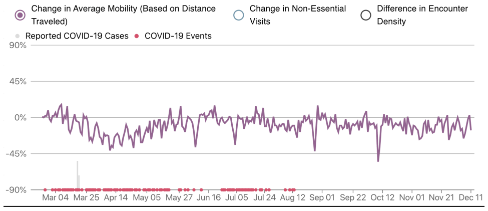

(b) West Baton Rouge Average Mobility

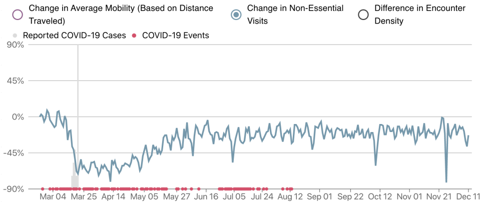

(c) East Baton Rouge Non-Essential Visits

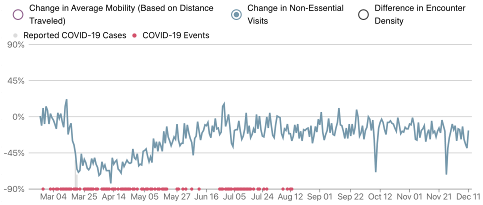

(d) West Baton Rouge Non-Essential Visits

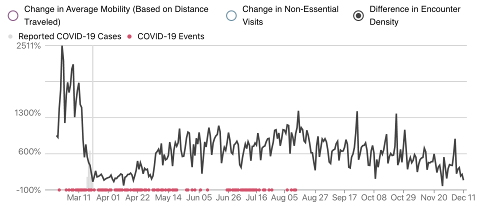

(e) East Baton Rouge Encounter Density

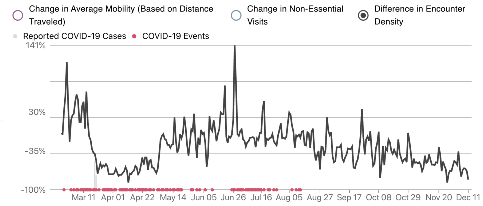

(f) West Baton Rouge Encounter Density

Fig S3. Metrics for adherence to social distancing guidelines in East and West Baton Rouge counties, which make up the entire parish, as provided by Unacast's dashboard. The most extreme drops in all three metrics occurred after the stay at home order was implemented.

Yearly Crime Trends Below are the crime dynamics from Chicago, Baltimore, and Baton Rouge from January of 2017 through October of 2020 (which was the last completed month of data collection at the time of manuscript submission). The plots show that 2020 has been a deviation from the expected crime dynamics, based on previous years. They also show that the steepest reductions in crime occurred soon after the stay at home orders were implemented.

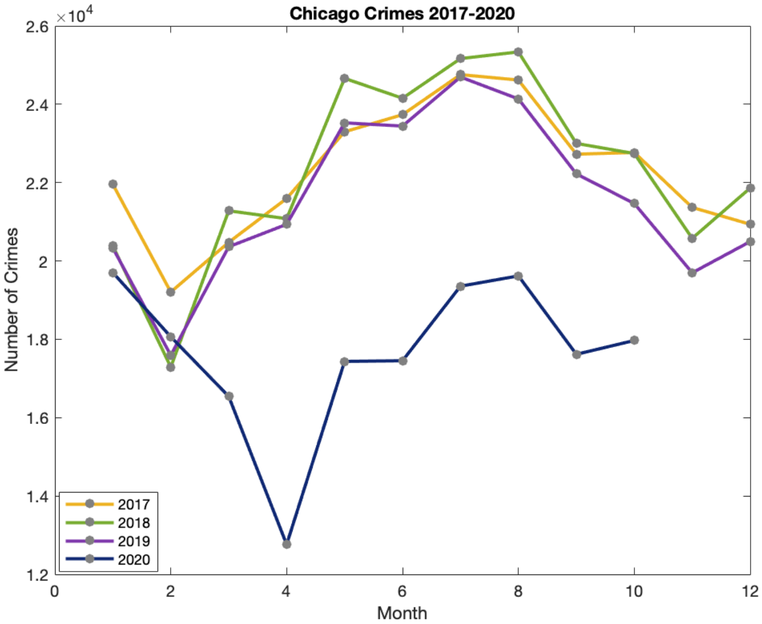

Fig S4. Chicago Crimes January 2017 - October 2020. The 2020 crime dynamics show shifts from the previous three years.

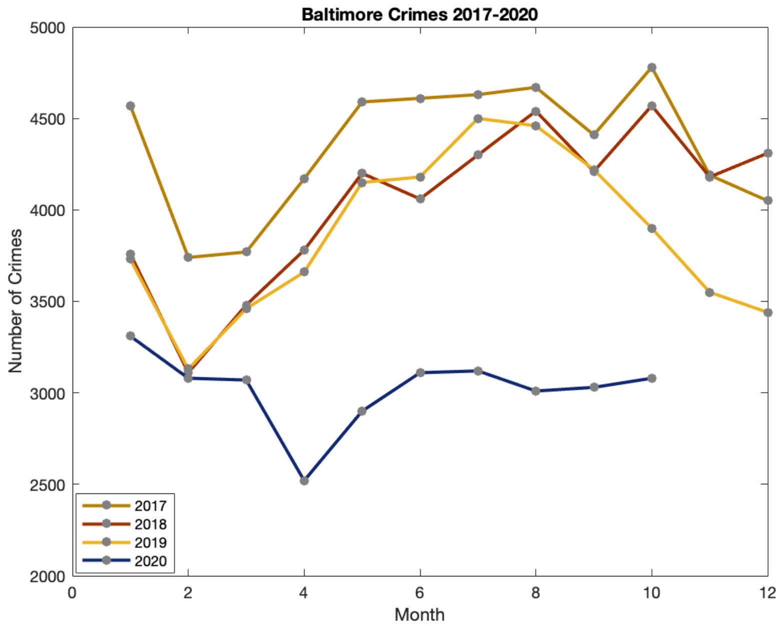

Fig S5. Baltimore Crimes January 2017 - October 2020. The 2020 crime dynamics show shifts from the previous three years.

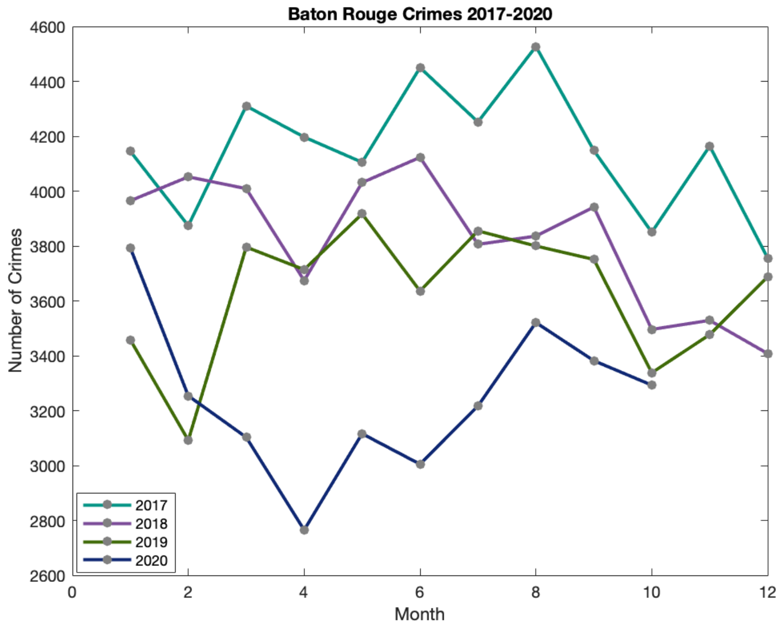

Fig S6. Baton Rouge Crimes January 2017 - October 2020. The 2020 crime dynamics show shifts from the previous three years.

Crime Type Descriptions The datasets publicly available from Chicago, Baltimore, and Baton Rouge differ in what items are provided for analysis. The means of collecting, defining, and reporting crimes can differ, which makes direct comparisons regarding crime types difficult. Below are descriptions of the specific crime types used in this analysis.

	Crime Type	Description
	**Chicago**	
	Gun Crimes	All crimes which involve the use of a firearm, based on secondary descriptions.
P	Arson	To unlawfully and intentionally damage or attempt to damage any real or personal property by fire or incendiary device.
I	Assault	An unlawful physical attack by one person upon another. Can be simple or aggravated.
I	Battery	Intentionally and knowingly without legal justification and by any means causes bodily harm to an individual or makes physical contact of an insulting or provoking nature with an individual. Can be simple or aggravated.
P	Burglary	The unlawful entry into a building ot other structure with the intent to commit a felony or theft.
P	Criminal Damage	To willfully or maliciously destroy, damage, deface, or otherwise injure real or personal property without the consent of the owner or the person having custody or control of it.
I	Criminal Sexual Assault	Any sexual act directed against another person, forcibly and/or against that person's will or not forcibly against the person's will in instances where the victim is incapable of giving consent.
P	Criminal Trespass	Violation of the laws and ordinances regarding land, airports, vehicles, residences, and state supplied land
I	Homicide	The killing of one human being by another.
S	Interference with Public Officer	Any behavior that tends to disturb the public peace or decorum, scandalize the community, or shock the public sense of morality involving a public officer.
S	Narcotics	The violation of laws prohibiting the production, distribution, and/or use of certain controlled substances and the equipment or devices utilized in their preparation and/or use.
S	Other Offense	The violation of miscellaneous laws or ordinances.
S	Public Peace Violation	Any behavior that tends to disturb the public peace.
P	Robbery	The taking or attempting to take anything of value under confrontational circumstances from the control, custody, or care of another person by force or threat of force or violence and/or by putting the victim in fear of immediate harm.
I	Sex Offense	The violation of laws prohibiting offenses against chastity, common decency, morals, and the like such as: adultery and fornication, bigamy, indecent exposure, and indecent liberties.
P	Theft	The unlawful taking, carrying, leading, or riding away of property from the possession or constructive possession of another person.
P	Weapons Violation	The violation of laws or ordinances prohibiting the manufacture, sale, purchase, transportation, concealment, or use of firearms, cutting instruments, explosives, incendiary devices, or other deadly weapons.
	**Baltimore**	
	Gun Crimes	All crimes which involve the use of a firearm, based on secondary descriptions.
I	Assault	An unlawful physical attack by one person upon another. Can be simple or aggravated.
P	Arson	To unlawfully and intentionally damage or attempt to damage any real or personal property by fire or incendiary device.
P	Auto Theft	The theft of an automobile.
P	Burglary	The unlawful entry into a building ot other structure with the intent to commit a felony or theft.
I	Homicide	The killing of one human being by another.
P	Larceny	The unlawful taking, carrying, leading, or riding away of property from the possession or constructive possession of another person.
I	Rape	Non-consensual vaginal intercourse. Does not include other sex offenses.
P	Robbery	The taking or attempting to take anything of value under confrontational circumstances from the control, custody, or care of another person by force or threat of force or violence and/or by putting the victim in fear of immediate harm.
I	Shooting	Intentional discharge of a firearm or other projectile weapon.
	**Baton Rouge**	
I	Assault	An unlawful physical attack by one person upon another. Can be simple or aggravated.
I	Battery	Intentionally and knowingly without legal justification and by any means causes bodily harm to an individual or makes physical contact of an insulting or provoking nature with an individual. Can be simple or aggravated.
P	Burglary	The unlawful entry into a building ot other structure with the intent to commit a felony or theft.
P	Criminal Damage	To willfully or maliciously destroy, damage, deface, or otherwise injure real or personal property without the consent of the owner or the person having custody or control of it.
P	Firearm	Unlawful use of a firearm
I	Homicide	The killing of one human being by another.
S	Juvenile	The act of participating in unlawful behavior as a minor or individual younger than the statutory age of majority
S	Narcotics	The violation of laws prohibiting the production, distribution, and/or use of certain controlled substances and the equipment or devices utilized in their preparation and/or use.
S	Nuisance	A human activity or a physical condition that is harmful or offensive to others and gives rise to a cause of action.
S	Other	The violation of miscellaneous laws or ordinances.
P	Robbery	The taking or attempting to take anything of value under confrontational circumstances from the control, custody, or care of another person by force or threat of force or violence and/or by putting the victim in fear of immediate harm.
I	Sexual Assault	An act of violence in which a person subjects a victim to contact of a sexual nature against the victim's will
P	Theft	The unlawful taking, carrying, leading, or riding away of property from the possession or constructive possession of another person.
S	Vice	A behavior considered immoral, criminal, rue or degrading in the associated society

Table S2. Descriptions of the various crime types from each city analyzed in this study. The crime categories are denoted on the left hand side of this table. Property crimes are denoted with (P), statutory crimes are denoted with (S), and interpersonal crimes are denoted with (I).

Further Methods

Moving Averages Analysis The underlying purpose of the moving average is to track the trend determination of the given time series data. The simple moving average calculation is a common average of the previous n data points in time series data:

$$SMA = \frac{P_M + P_{M-1} + ... + P_{M-(n-1)}}{n},$$

where P_M represents the data point value at time M and n represents the number of data points used in the calculation. There is thus a window over which the averaging occurs, denoted by k. For this study, we use $k = 5$ to show the averages of the data points over a five day period of time.

Further Results

Changes in Crime Categories Pre- and Post-Stay-at-Home Orders In the main text, we split crimes into three categories: personal (P), statutory (S), and interpersonal (I). The plots below sum the number of each crime type during each time period, average the number of those crime categories that occur on a daily basis, and compare the two. The first four plots compare pre- and post-stay-at-home order crimes in Chicago. In the first three plots, all crime categories increase between the first and second time periods. In the fourth plot, which shows 2020, all three crime categories decrease between time periods.

For Baltimore, there are only two crime categories (property and interpersonal) due to the available data. Both crime categories decrease between time periods. For Baton Rouge, all three crime categories decline between the two time periods. It is worth noting that in all three cities, the crime categories displaying larger declines are statutory and/or property crimes, rather than interpersonal crimes.

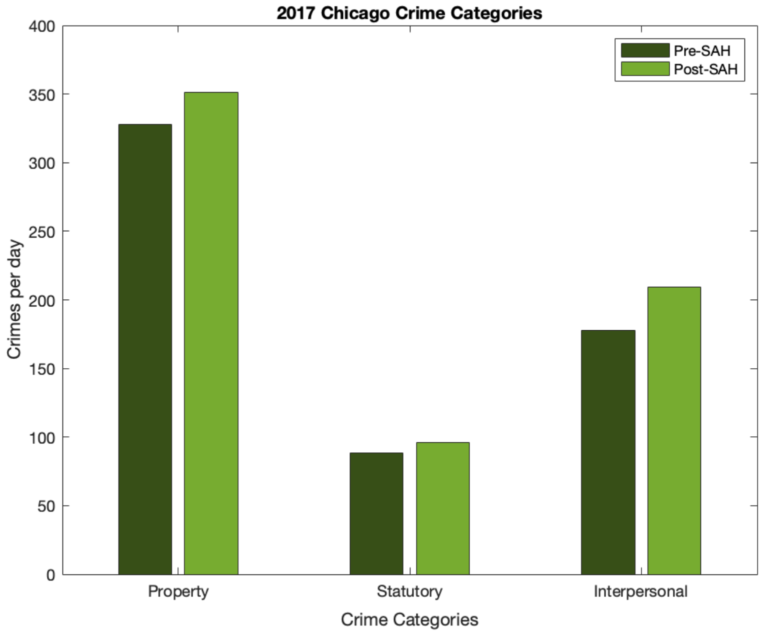

Fig S7. Crime categories from 2017 comparing the time period pre- and post-stay-at-home (SAH) in Chicago. In all three crime categories, average crimes increased between the pre- and post-stay-at-home time periods.

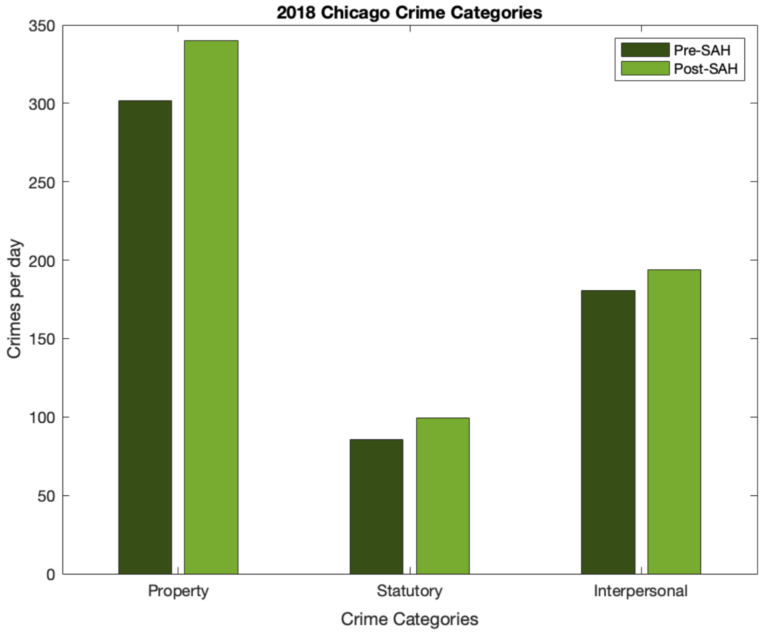

Fig S8. Crime categories from 2018 comparing the time period pre- and post-stay-at-home (SAH) in Chicago. In all three crime categories, average crimes increased between the pre- and post-stay-at-home time periods.

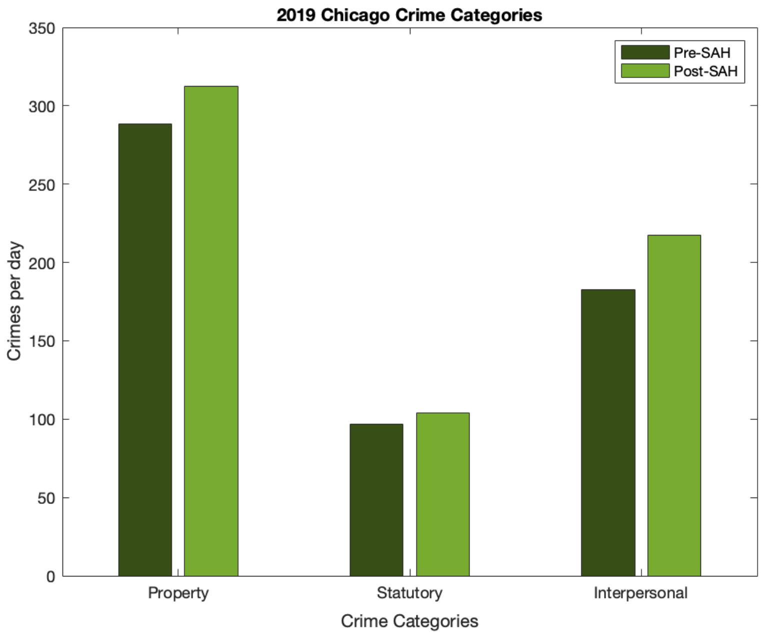

Fig S9. Crime categories from 2019 comparing the time period pre- and post-stay-at-home (SAH) in Chicago. In all three crime categories, average crimes increased between the pre- and post-stay-at-home time periods.

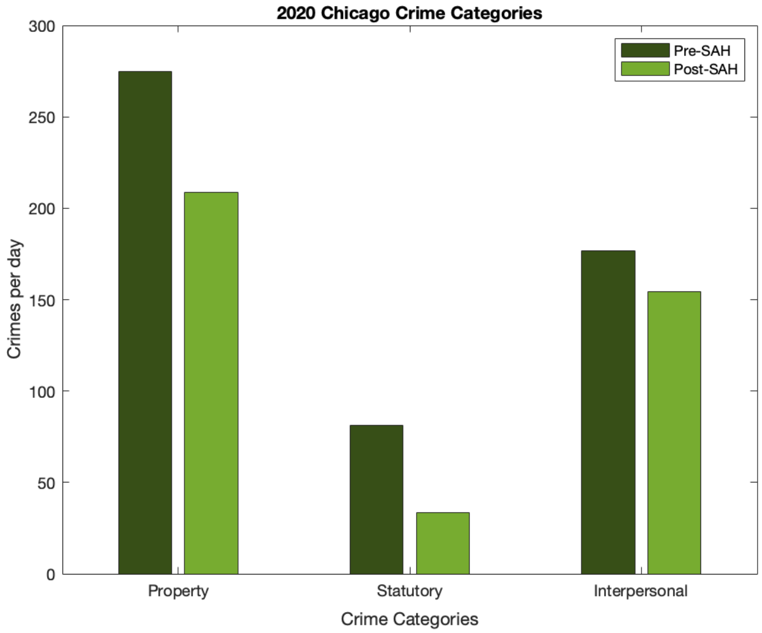

Fig S10. Crime categories from 2020 comparing the time period pre- and post-stay-at-home (SAH) in Chicago. In all three crime categories, average crimes decreased between the pre- and post-stay-at-home time periods, differing from the previous years.

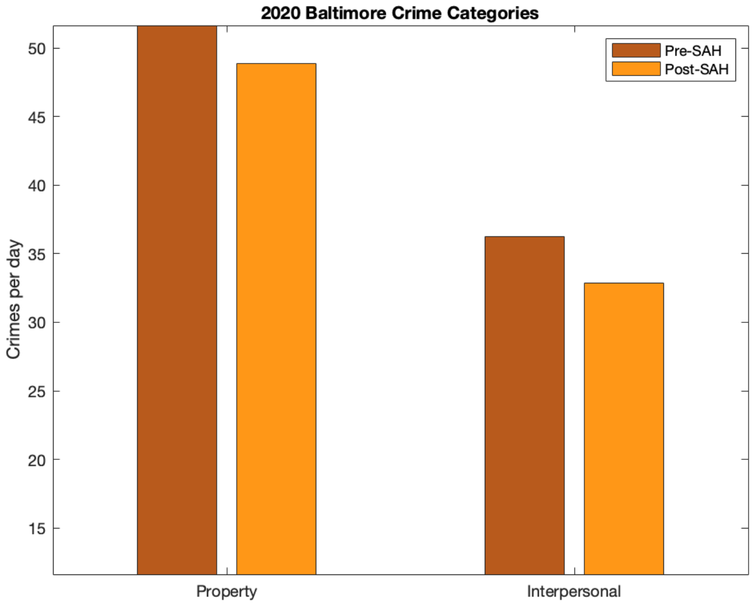

Fig S11. Crime categories from 2020 comparing the time period pre- and post-stay-at-home (SAH) in Baltimore. In all three crime categories, average crimes decreased between the pre- and post-stay-at-home time periods.

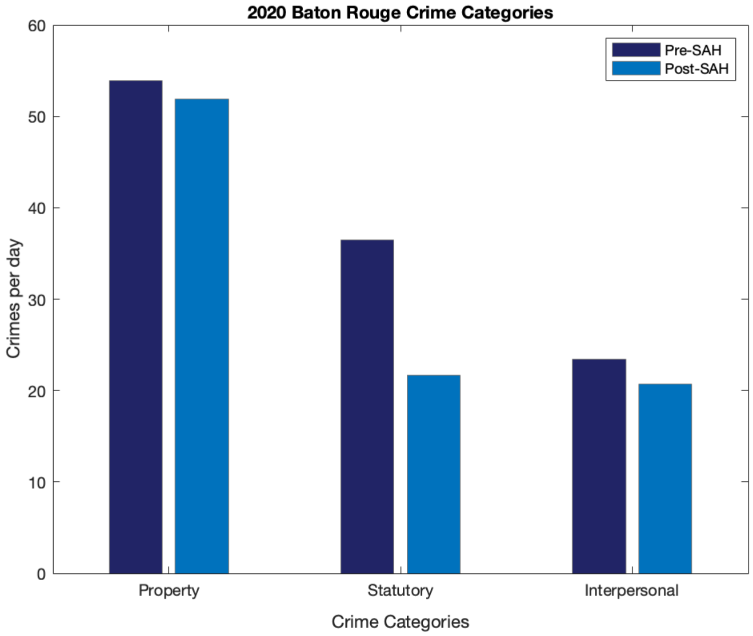

Fig S12. Crime categories from 2020 comparing the time period pre- and post-stay-at-home (SAH) in Baton Rouge. In all three crime categories, average crimes decreased between the pre- and post-stay-at-home time periods.

Chicago Year in Year Analyses To determine whether the patterns observed in 2020 are unique to this year and not reflected in past years, we perform year in year analyses over different time periods. In the main text, we find that 2017, 2018, and 2019 differ significantly from 2020. We test the three time periods (pre-COVID, state of emergency, and stay at home) against the early (January 1 - March 31) dataset of 2019 to also confirm that seasonality is not affecting these observations. We then compare each of the three time periods between the previous three years (2017, 2018, and 2019) to 2020. The results are as shown in tables S3-S17 and figures S4, S7-S10, and S13.

Early 2019 Crime Types (μ, σ)	2019 Crime Types - Pre-COVID(μ, σ)	p-value	ci	t-score	STDev	Percent Change
Total Crimes (650, 84.2)	Total Crimes (640, 95.0)	0.519	[-18.9, 37.3]	0.646	88.9	-1.54%
Gun Crimes (33.6, 9.37)	Gun Crimes (32.8, 10.1)	0.630	[-2.32, 3.82]	0.482	9.72	-2.38
Arson (0.872, 0.845)	Arson (0.821, 0.852)	0.705	[-0.216, 0.319]	0.380	0.875	-5.85%
Assault (50.8, 9.89)	Assault (48.4, 9.36)	0.119	[-0.632, 5.48]	1.57	9.67	-4.72%
Battery (122, 22.5)	Battery (116, 21.2)	0.119	[-1.43, 12.4]	1.57	22.0	-4.92%
Burglary (23.7, 7.37)	Burglary (24.3, 7.55)	0.621	[-2.94, 1.76]	-0.496	7.45	2.53%
Criminal Damage (61.7, 13.8)	Criminal Damage (59.2, 15.1)	0.279	[-2.04, 7.00]	1.09	14.3	-4.05%
Criminal Sexual Assault (3.81, 2.44)	Criminal Sexual Assault (3.79, 2.56)	0.965	[-0.769, 0.804]	0.044	2.49	-0.525%
Criminal Trespass (17.5, 5.02)	Criminal Trespass (17.6, 5.42)	0.915	[-1.73, 1.55]	-0.107	5.19	0.571%
Homicide (0.989, 1.05)	Homicide (0.821, 0.984)	0.305	[-0.155, 0.492]	1.03	1.02	-17.0%
Interference with Public Officer (3.40, 1.65)	Interference with Public Officer (3.48, 1.77)	0.788	[-0.611, 0.465]	-0.269	1.70	2.35%
Narcotics (44.1, 11.0)	Narcotics (44.5, 12.4)	0.861	[-3.99, 3.34]	-0.175	11.6	0.907%
Other Offense (47.8, 9.80)	Other Offense (48.0, 10.6)	0.912	[-3.38, 3.03]	-0.110	10.1	0.418%
Public Peace Violation (3.81, 2.18)	Public Peace Violation (3.31, 1.92)	0.138	[-0.160, 1.15]	1.49	2.07	-13.1%
Robbery (18.4, 5.59)	Robbery (19.4, 5.88)	0.276	[-2.80, 0.805]	-1.09	5.71	5.43%
Sex Offense (3.28, 2.37)	Sex Offense (3.28, 2.58)	0.986	[-0.782, 0.768]	-0.018	2.46	0.214%
Theft (149, 23.7)	Theft (149, 26.2)	0.994	[-7.86, 7.80]	-0.00710	24.8	0.019%
Weapons Violation (14.5, 5.93)	Weapons Violation (14.0, 6.34)	0.607	[-1.42, 2.43]	0.515	6.10	-3.45%

Table S3. Comparisons of the Pre-COVID time period in 2019 as a control to rule out seasonality as a mechanism for crime dynamics seen in 2020. The Pre-COVID time period is from 01/01/2020 - 03/08/2020. The Bonferroni correction with $n = 18$ produces a corrected significance value of $\alpha = 0.0027$ and the degrees of freedom are 159. There are no crime types that show significant differences between these two time periods.

Early 2019 Crime Types	2019 Crime Types - State of Emergency	p-value	ci	t-score	STDev	Percent Change
Total Crimes (650, 84.3)	Total Crimes (666, 50.6)	0.499	[-66.4, 32.6]	-0.678	81.4	2.46%
Gun Crimes (33.6, 9.37)	Gun Crimes (35.2, 5.97)	0.571	[-7.10, 3.93]	-0.569	9.07	4.55%
Arson (0.872, 0.845)	Arson (1.08, 1.08)	0.433	[-0.742, 0.320]	-0.788	0.874	19.3%
Assault (50.8, 9.89)	Assault (58.4, 8.71)	0.013	[-13.5, -1.64]	-2.53	9.77	13.0%
Battery (122, 22.5)	Battery (135, 24.4)	0.061	[-26.9, 0.637]	-1.89	22.7	9.63%
Burglary (23.7, 7.37)	Burglary (21.9, 5.99)	0.417	[-2.59, 6.21]	0.815	7.24	-8.22%
Criminal Damage (61.7, 13.8)	Criminal Damage (69.5, 5.70)	0.056	[-15.8, 0.213]	-1.93	13.1	11.2%
Criminal Sexual Assault (3.81, 2.44)	Criminal Sexual Assault (3.75, 2.01)	0.937	[-1.40, 1.52]	0.080	2.40	-1.6%
Criminal Trespass (17.5, 5.02)	Criminal Trespass (18.1, 3.87)	0.689	[-3.59, 2.38]	-0.401	4.91	3.31%
Homicide (0.989, 1.05)	Homicide (0.917, 0.900)	0.820	[-0.558, 0.703]	0.229	1.04	-7.29%
Interference with Public Officer (3.40, 1.65)	Interference with Public Officer (3.58, 1.38)	0.720	[-1.17, 0.810]	-0.359	1.63	5.03%
Narcotics (44.1, 11.0)	Narcotics (42.8, 6.75)	0.689	[-5.16, 7.77]	0.401	10.6	-3.04%
Other Offense (47.8, 9.80)	Other Offense (45.8, 6.52)	0.506	[-3.84, 7.72]	0.667	9.51	-4.37%
Public Peace Violation (3.81, 2.18)	Public Peace Violation (5.08, 2.31)	0.061	[-2.61, 0.060]	-1.89	2.20	25.0%
Robbery (18.4, 5.59)	Robbery (15.6, 3.50)	0.092	[-0.466, 6.11]	1.70	5.21	-17.9%
Sex Offense (3.28, 2.37)	Sex Offense (3.33, 2.31)	0.938	[-1.49, 1.38]	-0.078	2.36	1.50%
Theft (149, 23.7)	Theft (144, 17.0)	0.503	[-9.29, 18.8]	0.672	23.1	-3.47%
Weapons Violation (14.5, 5.93)	Weapons Violation (15.6, 3.87)	0.524	[-4.62, 2.37]	-0.639	5.75	7.05%

Table S4. Comparisons of the Pre-COVID time period in 2019 as a control to rule out seasonality as a mechanism for crime dynamics seen in 2020. The SOE time period represents when the state of emergency in Chicago was announced and spans from 03/09/2020 - 03/20/2020. The Bonferroni correction with $n = 18$ produces a corrected significance value of $\alpha = 0.0027$ and the degrees of freedom are 104. There are no crime types that show significant differences between these two time periods.

**Early 2019 Crime Types**	**2019 Crime Types - Stay at Home**	**p-value**	**ci**	**t-score**	**STDev**	**Percent Change**
Total Crimes (650, 84.3)	Total Crimes (677, 33.3)	0.216	[-71.3, 16.3]	-1.24	79.5	3.99%
Gun Crimes (33.6, 9.37)	Gun Crimes (35.7, 7.56)	0.415	[-7.13, 2.97]	-0.818	9.16	5.88%
Arson (0.872, 0.845)	Arson (0.933, 0.594)	0.789	[-0.511, 0.389]	-0.269	0.817	6.54%
Assault (50.8, 9.89)	Assault (55.6, 8.68)	0.082	[-10.1, 0.608]	-1.76	9.74	8.63%
Battery (122, 22.5)	Battery (136, 16.2)	0.022	[-26.1, -2.09]	-2.33	21.7	10.3%
Burglary (23.7, 7.37)	Burglary (22.5, 7.58)	0.564	[-2.89, 5.267]	0.579	7.40	-5.33%
Criminal Damage (61.7, 13.8)	Criminal Damage (66.6, 7.52)	0.184	[-12.1, 2.35]	-1.34	13.1	7.36%
Criminal Sexual Assault (3.81, 2.44)	Criminal Sexual Assault (3.93, 2.37)	0.854	[-1.47, 1.22]	-0.185	2.43	3.31%
Criminal Trespass (17.5, 5.02)	Criminal Trespass (16.6, 4.01)	0.521	[-1.82, 3.58]	0.645	4.90	-5.42%
Homicide (0.989, 1.05)	Homicide (1.80, 1.15)	0.007	[-1.40, 0.224]	-2.74	1.07	45.0%
Interference with Public Officer (3.40, 1.65)	Interference with Public Officer (2.93, 1.28)	0.295	[-0.417, 1.36]	1.05	1.61	-16.4%
Narcotics (44.1, 11.0)	Narcotics (43.7, 6.30)	0.890	[-5.38, 6.19]	0.139	10.5	-0.915%
Other Offense (47.8, 9.80)	Other Offense (48.5, 8.36)	0.778	[-6.06, 4.55]	-0.283	9.62	1.44%
Public Peace Violation (3.81, 2.18)	Public Peace Violation (5, 2.45)	0.056	[-2.41, 0.031]	-1.93	2.22	23.8%
Robbery (18.4, 5.59)	Robbery (16.2, 4.26)	0.148	[-0.791, 5.20]	1.46	5.43	-13.6%
Sex Offense (3.28, 2.37)	Sex Offense (3.20, 1.32)	0.903	[-1.17, 1.32]	0.122	2.26	-2.5%
Theft (149, 23.7)	Theft (153, 15.5)	0.563	[-16.3, 8.90]	-0.581	22.8	3.25%
Weapons Violation (14.5, 5.93)	Weapons Violation (15.8, 5.29)	0.412	[-4.57, 1.88]	-0.826	5.85	8.23%

Table S5. Comparisons of the Pre-COVID time period in 2019 as a control to rule out seasonality as a mechanism for crime dynamics seen in 2020. The SAH time period represents the time period when the stay at home order was put in place and spans from 03/21/2020 - 04/04/2020. The Bonferroni correction with $n = 18$ produces a corrected significance value of $\alpha = 0.0027$ and the degrees of freedom are 107. There are no crime types that show significant differences between these two time periods.

**Early 2020 Crime Types**	**2020 Crime Types - Pre-COVID**	**p-value**	**ci**	**t-score**	**STDev**	**Percent Change**
**Total Crimes (575, 90.6)**	**Total Crimes (617, 51.3)**	7.21×10^{-4}	[-66.1, -178.0]	-3.45	76.7	6.81%
Gun Crimes (36.4, 9.38)	Gun Crimes (37.5, 9.74)	0.487	[-4.04, 1.94]	-0.696	9.53	2.93%
Arson (0.884, 1.07)	Arson (0.882, 1.10)	0.991	[-0.338, 0.342]	0.011	1.08	-0.227%
Assault (47.4, 8.02)	Assault (48.9, 7.32)	0.242	[-3.87, 0.984]	-1.17	7.74	3.07%
Battery (115, 21.7)	Battery (117, 22.4)	0.505	[-9.22, 4.56]	-0.668	22.0	1.17%
Burglary (20.6, 5.16)	Burglary (21.9, 5.04)	0.121	[-2.87, 0.339]	-1.56	5.11	5.94%
Criminal Damage (59.1, 12.4)	Criminal Damage (59.5, 12.9)	0.836	[-4.37, 3.54]	-0.208	12.6	0.572%
Criminal Sexual Assault (3.73, 2.47)	Criminal Sexual Assault (4.31, 2.52)	0.143	[-1.36, 0.199]	-1.47	2.49	13.5%
Criminal Trespass (16.1, 5.70)	Criminal Trespass (18.1, 4.83)	0.020	[-3.69, -0.324]	-2.36	5.36	11.0%
Homicide (1.08, 1.04)	Homicide (1.24, 1.12)	0.377	[-0.488, 0.186]	-0.886	1.07	12.9%
Interference with Public Officer (3.32, 2.19)	Interference with Public Officer (4.04, 1.86)	0.027	[-1.37, -0.083]	-2.23	2.06	17.8%
**Narcotics (31.0, 13.6)**	**Narcotics (37.3, 6.89)**	6.67×10^{-4}	[-9.76, -2.68]	-3.47	11.3	16.9%
Other Offense (39.4, 9.98)	Other Offense (43.4, 7.65)	0.009	[-6.69, -0.989]	-2.66	9.08	9.22%
Public Peace Violation (2.87, 2.06)	Public Peace Violation (3.22, 2.04)	0.288	[-0.990, 0.296]	-1.07	2.05	10.9%
Robbery (20.4, 6.11)	Robbery (22.1, 5.62)	0.075	[-3.54, 0.172]	-1.79	5.91	7.69%
Sex Offense (2.78, 1.65)	Sex Offense (2.93, 1.59)	0.568	[-0.656, 0.361]	-0.573	1.62	5.12%
**Theft (130, 28.9)**	**Theft (143, 18.2)**	8.61×10^{-4}	[-21.3, -5.64]	-3.40	25.0	9.09%
Weapons Violation (15.6, 5.80)	Weapons Violation (16.1, 6.33)	0.603	[-2.39, 1.39]	-0.521	6.03	3.11%

Table S6. Comparisons of differing time periods in 2020 for each crime type. The Pre-COVID time period spans from from 01/01/2020 - 03/08/2020. The Bonferroni correction with $n = 18$ produces a corrected significance value of $\alpha = 0.0027$ and the degrees of freedom are 161. There are a number of crime types that show significant differences between the two time periods.

**Early 2020 Crime Types**	**2020 Crime Types - State of Emergency**	**p-value**	**ci**	**t-score**	**STDev**	**Percent Change**
Total Crimes (575, 90.6)	Total Crimes (538, 59.2)	0.171	[-16.3, 90.5]	1.38	87.8	-
Gun Crimes (36.4, 9.78)	Gun Crimes (34.6, 7.60)	0.514	[-3.74, 7.44]	0.655	9.21	-5.20%
Arson (0.884, 1.07)	Arson (0.750, 1.06)	0.683	[-0.515, 0.784]	0.410	1.07	-17.9%
Assault (47.4, 8.02)	Assault (49.3, 7.39)	0.452	[-6.67, 2.99]	-0.755	7.96	3.85%
Battery (115, 21.7)	Battery (120, 17.9)	0.436	[-18.1, 7.83]	-0.783	21.3	4.17%
Burglary (20.6, 5.16)	Burglary (18.5, 4.50)	0.184	[-1.01, 5.19]	1.34	5.10	-11.4%
Criminal Damage (59.1, 12.4)	Criminal Damage (60, 10.6)	0.807	[-8.33, 6.49]	-0.245	12.2	1.5%
Criminal Sexual Assault (3.73, 2.47)	Criminal Sexual Assault (2.83, 2.04)	0.233	[-0.584, 2.37]	1.20	2.43	-31.8%
Criminal Trespass (16.1, 5.70)	Criminal Trespass (13.9, 5.21)	0.214	[-1.27, 5.60]	1.25	5.66	-15.8%
Homicide (1.08, 1.04)	Homicide (0.667, 0.651)	0.178	[-0.193, 1.03]	1.36	1.01	-61.9%
Interference with Public Officer (3.32, 2.19)	Interference with Public Officer (3.00, 1.91)	0.634	[-0.997, 1.63]	0.477	2.16	-10.7%
Narcotics (31.0, 13.6)	Narcotics (28.3, 10.3)	0.508	[-5.36, 10.8]	0.664	13.3	-9.54%
Other Offense (39.4, 9.98)	Other Offense (36.0, 8.49)	0.256	[-2.53, 9.42]	1.14	9.83	-9.44%
Public Peace Violation (2.87, 2.06)	Public Peace Violation (2.67, 2.53)	0.750	[-1.08, 1.49]	0.320	2.11	-6.97%
Robbery (20.4, 6.11)	Robbery (16.7, 5.37)	0.045	[0.090, 7.42]	2.03	6.03	-22.2%
Sex Offense (2.78, 1.65)	Sex Offense (2.83, 2.04)	0.917	[-1.08, 0.973]	-0.105	1.69	1.77%
Theft (130, 28.9)	Theft (113, 20.5)	0.056	[-0.452, 33.7]	1.93	28.1	-15.0%
Weapons Violation (15.6, 5.80)	Weapons Violation (14.4, 3.70)	0.497	[-2.24, 4.59]	0.681	5.62	-8.33%

Table S7. Comparisons of differing time periods in 2020 for each crime type. The state of emergency time period in Chicago spans from 03/09/2020 - 03/20/2020. The Bonferroni correction with $n = 18$ produces a corrected significance value of $\alpha = 0.0027$ and the degrees of freedom are 105. There are no crime types that show significant differences between these two time periods.

Early 2020 Crime Types	2020 Crime Types - Stay at Home	p-value	ci	t-score	STDev	Percent Change
**Total Crimes (575, 90.6)**	**Total Crimes (415, 47.8)**	9.26×10^{-10}	[113, 208]	6.71	86.3	-38.6%
Gun Crimes (36.4, 9.38)	Gun Crimes (33.1, 8.42)	0.203	[-1.80, 8.40]	1.28	9.26	-9.97%
Arson (0.884, 1.07)	Arson (1.00, 1.00)	0.695	[-0.701, 0.469]	-0.393	1.06	11.6%
**Assault (47.4, 8.02)**	**Assault (39.4, 7.14)**	4.13×10^{-4}	[3.65, 12.4]	3.65	7.91	-20.3%
Battery (115, 21.7)	Battery (100, 15.2)	0.013	[3.11, 26.2]	2.52	20.9	-15.0%
Burglary (20.6, 5.16)	Burglary (16.5, 3.52)	0.004	[1.31, 6.80]	2.93	4.98	-24.8%
Criminal Damage (59.1, 12.4)	Criminal Damage (56.5, 11.5)	0.444	[-4.14, 9.37]	0.768	12.3	-4.40%
Criminal Sexual Assault (3.73, 2.47)	Criminal Sexual Assault (1.80, 1.08)	0.004	[0.638, 3.22]	2.96	2.34	-51.7%
**Criminal Trespass (16.1, 5.7)**	**Criminal Trespass (8.73, 2.05)**	3.07×10^{-6}	[4.39, 10.3]	4.92	5.37	-45.8%
Homicide (1.08, 1.04)	Homicide (0.733, 0.704)	0.210	[-0.201, 0.902]	1.26	1.00	-32.1%
**Interference with Public Officer (3.32, 2.19)**	**Interference with Public Officer (0.267, 0.458)**	4.85×10^{-7}	[1.92, 4.18]	5.36	2.05	-92.0%
**Narcotics (31.0, 13.6)**	**Narcotics (5.00, 2.56)**	3.42×10^{-11}	[19.0, 33.0]	7.38	12.7	-83.9%
**Other Offense (39.4, 9.98)**	**Other Offense (24.8, 4.54)**	1.83×10^{-7}	[9.44, 19.9]	5.58	9.45	-37.1%
Public Peace Violation (2.87, 2.06)	Public Peace Violation (1.47, 0.916)	0.011	[0.334, 2.48]	2.60	1.95	-48.8%
Robbery (20.4, 6.11)	Robbery (15.8, 5.28)	0.007	[1.31, 7.93]	2.77	6.01	-22.5%
Sex Offense (2.78, 1.65)	Sex Offense (2.07, 1.49)	0.118	[-0.183, 1.61]	1.58	1.63	-25.5%
**Theft (130, 28.9)**	**Theft (82.0, 11.0)**	6.04×10^{-9}	[32.8, 62.8]	6.32	27.2	-36.9%
Weapons Violation (15.6, 5.80)	Weapons Violation (14.3, 4.41)	0.401	[-1.79, 4.43]	0.844	5.64	-8.33%

Table S8. Comparisons of stay at home time periods in 2020 for each crime type. The stay at home order spans from 03/21/2020 - 03/31/2020. The Bonferroni correction with $n = 18$ produces a corrected significance value of $\alpha = 0.0027$ and the degrees of freedom are 108. There are a number of crime types that show significant differences between the two time periods.

2019 Crime Types - Pre-COVID	2020 Crime Types - Pre-COVID	p-value	ci	t-score	STDev	Percent Change
Total Crimes (640, 95.1)	Total Crimes (617, 51.3)	0.083	[-3.03, 48.9]	1.75	76.2	-3.59%
Gun Crimes (32.8, 10.2)	Gun Crimes (37.5, 9.74)	0.008	[-8.04, -1.26]	-2.71	9.96	14.3%
Arson (0.821, 0.852)	Arson (0.882, 1.10)	0.717	[-0.397, 0.274]	-0.363	0.985	7.43%
Assault (48.4, 9.36)	Assault (48.9, 7.32)	0.764	[-3.29, 2.423]	-0.301	8.394	1.03%
Battery (116, 21.2)	Battery (117, 22.4)	0.799	[-8.38, 6.47]	-0.255	21.8	0.862%
Burglary (24.3, 7.55)	Burglary (21.9, 5.04)	0.027	[0.278, 4.64]	2.23	6.41	-9.88%
Criminal Damage (59.2, 15.1)	Criminal Damage (59.5, 12.9)	0.914	[-5.03, 4.51]	-0.108	14.0	0.507%
Criminal Sexual Assault (3.79, 2.56)	Criminal Sexual Assault (4.31, 2.52)	0.238	[-1.38, 0.346]	-1.19	2.54	13.7%
Criminal Trespass (17.6, 5.42)	Criminal Trespass (18.1, 4.83)	0.556	[-2.27, 1.23]	-0.590	5.13	2.84%
Homicide (0.821, 0.984)	Homicide (1.23, 1.12)	0.024	[-0.774, -0.055]	-2.28	1.06	49.8%
Interference with Public Officer (3.48, 1.77)	Interference with Public Officer (4.04, 1.86)	0.072	[-1.18, 0.051]	-1.81	1.81	16.1%
**Narcotics (44.5, 12.4)**	**Narcotics (37.3, 6.89)**	5.15×10^{-5}	[3.80, 10.6]	4.18	10.0	-16.2%
Other Offense (48.0, 10.6)	Other Offense (43.3, 7.65)	0.004	[1.54, 7.82]	2.94	9.23	-9.79%
Public Peace Violation (3.31, 1.92)	Public Peace Violation (3.22, 2.04)	0.786	[-0.581, 0.767]	0.273	1.98	-2.72%
Robbery (19.4, 5.88)	Robbery (22.1, 5.62)	0.007	[-4.66, -0.742]	-2.73	5.75	13.9%
Sex Offense (3.28, 2.58)	Sex Offense (2.93, 1.59)	0.333	[-0.370, 1.08]	0.972	2.14	-10.7%
Theft (149, 26.2)	Theft (143, 18.2)	0.128	[-1.72, 13.6]	1.533	22.5	-4.03%
Weapons Violation (14.0, 6.34)	Weapons Violation (16.1, 6.33)	0.053	[-4.29, 0.023]	-1.96	6.33	15.0%

Table S9. Comparisons of 2019 crime types and 2020 crime types before protocols were put in place in response to COVID-19. The Bonferroni correction with $n = 18$ produces a corrected significance value of $\alpha = 0.0027$ and the degrees of freedom are 133. Narcotics is the only crime type showing significant declines between the two time periods.

2018 Crime Types - Pre-COVID	2020 Crime Types - Pre-COVID	p-value	ci	t-score	STDev	Percent Change
Total Crimes (644, 70.5)	Total Crimes (617, 51.3)	0.015	[5.22, 47.2]	2.47	61.6	-4.19%
Gun Crimes (35.2, 8.17)	Gun Crimes (37.5, 9.74)	0.147	[-5.33, 0.802]	-1.46	9.00	6.53%
Arson (0.806, 0.925)	Arson (0.882, 1.10)	0.663	[-0.423, 0.270]	-0.436	1.02	9.43%
Assault (48.0, 9.64)	Assault (48.9, 7.32)	0.563	[-3.76, 2.06]	-0.580	8.55	1.88%
Battery (116, 18.8)	Battery (117, 22.4)	0.792	[-7.97, 6.09]	-0.264	20.7	0.862%
**Burglary (27.5, 6.89)**	**Burglary (21.9, 5.04)**	2.36×10^{-7}	[3.60, 7.71]	5.45	6.03	-20.4%
Criminal Damage (62.2, 14.1)	Criminal Damage (59.5, 12.9)	0.252	[-1.93, 7.29]	1.15	13.5	-4.34%
Criminal Sexual Assault (3.78, 2.82)	Criminal Sexual Assault (4.31, 2.52)	0.249	[-1.44, 0.376]	-1.16	2.67	14.0%
Criminal Trespass (19.1, 4.01)	Criminal Trespass (18.1, 4.83)	0.180	[-0.481, 2.54]	1.35	4.44	-5.24%
Homicide (1.40, 1.19)	Homicide (1.24, 1.12)	0.402	[-0.227, 0.562]	0.841	1.16	-11.4%
Interference with Public Officer (3.16, 1.80)	Interference with Public Officer (4.04, 1.86)	0.006	[-1.50, -0.258]	-2.80	1.83	27.8%
Narcotics (36.1, 10.4)	Narcotics (37.3, 6.89)	0.470	[-4.11, 1.91]	-0.724	8.83	3.32%
Other Offense (44.6, 7.73)	Other Offense (43.3, 7.65)	0.338	[-1.35, 3.89]	0.962	7.69	-2.91%
Public Peace Violation (2.94, 1.59)	Public Peace Violation (3.22, 2.04)	0.375	[-0.903, 0.343]	-0.890	1.83	9.52%
**Robbery (25.5, 6.51)**	**Robbery (22.1, 5.62)**	0.001	[1.35, 5.49]	3.27	6.08	-13.3%
Sex Offense (2.52, 3.67)	Sex Offense (2.93, 1.59)	0.376	[-1.36, 0.555]	-0.833	2.82	16.3%
Theft (148, 23.8)	Theft (143, 18.2)	0.194	[-2.45, 11.9]	1.31	21.1	-3.38%
**Weapons Violation (12.1, 4.60)**	**Weapons Violation (16.1, 6.33)**	5.30×10^{-5}	[-5.87, -2.10]	-4.18	5.54	33.1%

Table S10. Comparisons of 2018 crime types and 2020 crime types before protocols were put in place in response to COVID-19. The Bonferroni correction with $n = 18$ produces a corrected significance value of $\alpha = 0.0027$ and the degrees of freedom are 133. Burglaries and weapons violations show significant differences, with burglaries declining and weapons violations increasing.

2017 Crime Types - Pre-COVID	2020 Crime Types - Pre-COVID	p-value	ci	t-score	STDev	Percent Change
**Total Crimes (694, 75.4)**	**Total Crimes (617, 51.3)**	1.85×10^{-10}	[54.6, 98.5]	6.91	64.4	-11.1%
Gun Crimes (39.1, 8.83)	Gun Crimes (37.5, 9.74)	0.327	[-1.59, 4.74]	0.983	9.30	-4.09%
Arson (1.31, 1.21)	Arson (0.884, 1.10)	0.032	[0.038, 0.824]	2.17	1.16	-32.5%
Assault (45.1, 7.59)	Assault (48.9, 7.32)	0.004	[-6.27, -1.19]	-2.91	7.46	6.65%
Battery (119, 20.3)	Battery (117, 22.4)	0.633	[-5.51, 9.03]	0.479	21.4	-1.68%
**Burglary (37.5, 11.4)**	**Burglary (21.9, 5.04)**	1.19×10^{-19}	[12.6, 18.6]	10.3	8.81	-41.6%
**Criminal Damage (74.5, 14.0)**	**Criminal Damage (59.5, 12.9)**	1.71×10^{-9}	[10.4, 19.6]	6.47	13.4	-20.1%
Criminal Sexual Assault (4.46, 3.98)	Criminal Sexual Assault (4.31, 2.52)	0.789	[-0.979, 1.29]	0.269	3.33	-3.36%
Criminal Trespass (18.6, 3.88)	Criminal Trespass (18.1, 4.83)	0.527	[-1.01, 1.97]	0.635	4.38	-2.69%
Homicide (1.67, 1.58)	Homicide (1.24, 1.12)	0.066	[-0.030, 0.902]	1.85	1.37	-25.7%
**Interference with Public Officer (2.85, 1.47)**	**Interference with Public Officer (4.04, 1.86)**	6.19×10^{-5}	[-1.76, -0.623]	-4.14	1.68	41.8%
Narcotics (33.6, 9.29)	Narcotics (37.3, 6.89)	0.011	[-6.41, -0.841]	-2.58	8.17	11.0%
**Other Offense (50.2, 9.43)**	**Other Offense (43.3, 7.65)**	7.53×10^{-6}	[3.96, 9.81]	4.66	8.58	-13.7%
Public Peace Violation (3.73, 2.16)	Public Peace Violation (3.22, 2.04)	0.160	[-0.205, 1.23]	1.41	2.10	-13.7%
**Robbery (30.4, 7.41)**	**Robbery (22.1, 5.62)**	1.77×10^{-12}	[6.08, 10.6]	7.35	6.57	-27.3%
Sex Offense (2.66, 4.33)	Sex Offense (2.93, 1.59)	0.630	[-1.38, 0.836]	-0.482	3.25	10.2%
**Theft (157, 20.2)**	**Theft (143, 18.2)**	5.18×10^{-9}	[7.48, 20.6]	4.24	19.2	-8.92%
**Weapons Violation (11.1, 4.47)**	**Weapons Violation (16.1, 6.33)**	5.95×10^{-7}	[-6.82, -3.09]	-5.25	5.48	45.0%

Table S11. Comparisons of 2017 crime types and 2020 crime types before protocols were put in place in response to COVID-19. The Bonferroni correction with $n = 18$ produces a corrected significance value of $\alpha = 0.0027$ and the degrees of freedom are 133. There are a number of crime types that show significant differences between the two time periods.

2019 Crime Types - Stay at Home	2020 Crime Types - Stay at Home	p-value	ci	t-score	STDev	Percent Change
**Total Crimes (677, 33.3)**	**Total Crimes (415, 47.8)**	1.41×10^{-16}	[232, 293]	17.5	41.2	-38.7%
Gun Crimes (35.7, 7.56)	Gun Crimes (33.1, 8.42)	0.393	[-3.45, 8.52]	0.868	8.00	-7.28%
Arson (0.933, 0.594)	Arson (1.00, 1.00)	0.826	[-0.682, 0.549]	-0.222	0.822	7.18%
**Assault (55.6, 8.68)**	**Assault (39.4, 7.14)**	5.63×10^{-6}	[10.3, 22.1]	5.59	7.94	-29.1%
**Battery (136, 16.2)**	**Battery (100, 15.2)**	1.00×10^{-6}	[23.9, 47.3]	6.23	15.7	-26.5%
Burglary (22.5, 7.58)	Burglary (16.5, 3.52)	0.010	[1.58, 10.4]	2.78	5.91	-26.7%
Criminal Damage (66.6, 7.52)	Criminal Damage (56.5, 11.5)	0.008	[2.86, 17.4]	2.85	9.72	-15.2%
Criminal Sexual Assault (2.93, 2.37)	Criminal Sexual Assault (1.80, 1.08)	0.004	[0.753, 3.51]	3.17	1.85	-38.6%
**Criminal Trespass (16.6, 4.01)**	**Criminal Trespass (8.73, 2.05)**	2.44×10^{-7}	[5.48, 10.3]	6.76	3.19	-47.4%
Homicide (1.80, 1.15)	Homicide (0.733, 0.704)	0.005	[0.355, 1.78]	3.07	0.951	-59.3%
**Interference with Public Officer (2.93, 1.28)**	**Interference with Public Officer (0.267, 0.458)**	2.82×10^{-8}	[1.95, 3.39]	7.60	0.961	-90.9%
**Narcotics (43.7, 6.30)**	**Narcotics (5.00, 2.56)**	3.03×10^{-19}	[35.1, 42.3]	22.1	4.81	-88.6%
**Other Offense (48.5, 8.36)**	**Other Offense (24.8, 4.54)**	2.03×10^{-10}	[18.7, 28.8]	9.67	6.73	-48.9%
**Public Peace Violation (5.00, 2.45)**	**Public Peace Violation (1.47, 0.916)**	1.47×10^{-5}	[2.15, 4.92]	5.23	1.85	-70.6%
Robbery (16.2, 4.26)	Robbery (15.8, 5.28)	0.821	[-3.19, 3.99]	0.228	4.80	-2.47%
Sex Offense (3.20, 1.32)	Sex Offense (2.07, 1.49)	0.036	[0.082, 2.19]	2.21	1.41	-35.3%
**Theft (153, 15.5)**	**Theft (82.0, 11.0)**	1.70×10^{-14}	[60.8, 80.9]	14.4	13.5	-46.4%
Weapons Violation (15.8, 5.29)	Weapons Violation (14.3, 4.42)	0.396	[-2.11, 5.18]	0.861	4.88	-9.49%

Table S12. Comparisons of 2019 crime types and 2020 crime types after the stay at home order was put in place. The Bonferroni correction with $n = 18$ produces a corrected significance value of $\alpha = 0.0027$ and the degrees of freedom are 28. There are a number of crime types that show significant differences between the two time periods.

2018 Crime Types - Stay at Home	2020 Crime Types - Stay at Home	p-value	ci	t-score	STDev	Percent Change
**Total Crimes (680, 35.2)**	**Total Crimes (415, 47.8)**	1.75×10^{-16}	[234, 297]	17.3	42.0	-39.0%
Gun Crimes (33.2, 8.06)	Gun Crimes (33.1, 8.42)	0.983	[-6.10, 6.23]	0.022	8.24	-0.301%
Arson (0.800, 1.01)	Arson (1.00, 1.00)	0.591	[-0.953, 0.553]	-0.544	1.01	25.0%
**Assault (49.2, 7.88)**	**Assault (39.4, 7.14)**	0.001	[4.17, 15.4]	2.78	8.06	-19.9%
Battery (121, 19.0)	Battery (100, 15.2)	0.003	[7.74, 33.5]	3.28	17.2	-17.4%
**Burglary (28.9, 3.50)**	**Burglary (16.5, 3.52)**	2.27×10^{-10}	[9.71, 15.0]	9.62	3.5	-42.9%
**Criminal Damage (77.3, 9.41)**	**Criminal Damage (56.5, 11.5)**	8.45×10^{-6}	[13.0, 28.7]	5.44	10.5	-26.9%
Criminal Sexual Assault (3.60, 1.99)	Criminal Sexual Assault (1.80, 1.08)	0.005	[0.601, 3.00]	3.07	1.60	-50.0%
**Criminal Trespass (19.1, 3.67)**	**Criminal Trespass (8.73, 2.05)**	2.88×10^{-10}	[8.12, 12.6]	9.51	2.98	-54.3%
Homicide (0.933, 1.10)	Homicide (0.733, 0.704)	0.558	[-0.491, 0.891]	0.593	0.923	-21.4%
**Interference with Public Officer (3.87, 1.77)**	**Interference with Public Officer (0.267, 0.458)**	2.56×10^{-8}	[2.63, 4.57]	7.64	1.29	-93.1%
**Narcotics (39.3, 7.70)**	**Narcotics (5.00, 2.56)**	1.96×10^{-16}	[30.0, 38.6]	16.4	5.74	-87.3
**Other Offense (49.3, 8.12)**	**Other Offense (24.8, 4.54)**	6.38×10^{-11}	[19.6, 29.4]	10.2	6.58	-49.7%
**Public Peace Violation (3.93, 1.79)**	**Public Peace Violation (1.47, 0.916)**	5.52×10^{-5}	[1.40, 3.53]	4.75	1.42	-62.6%
**Robbery (22.7, 5.04)**	**Robbery (15.8, 5.28)**	0.001	[3.01, 10.7]	3.64	5.16	-30.4%
Sex Offense (2.87, 1.92)	Sex Offense (2.07, 1.49)	0.213	[-0.485, 2.09]	1.28	1.72	-27.9%
**Theft (157, 10.3)**	**Theft (82.0, 11.0)**	9.08×10^{-18}	[67.3, 83.2]	19.4	10.6	-47.8%
Weapons Violation (11.6, 4.17)	Weapons Violation (14.3, 4.42)	0.100	[-5.88, 0.546]	-1.70	4.30	23.3%

Table S13. Comparisons of 2018 crime types and 2020 crime types after the stay at home order was put in place. The Bonferroni correction with $n = 18$ produces a corrected significance value of $\alpha = 0.0027$ and the degrees of freedom are 28. There are a number of crime types that show significant differences between the two time periods.

2017 Crime Types - Stay at Home	2020 Crime Types - Stay at Home	p-value	ci	t-score	STDev	Percent Change
**Total Crimes (703, 46.7)**	**Total Crimes (415, 47.8)**	4.39×10^{-16}	[253, 323]	16.7	47.3	-41.0%
Gun Crimes (36.5, 7.18)	Gun Crimes (33.1, 8.42)	0.253	[-2.52, 9.19]	1.17	7.82	-9.32%
Arson (1.00, 1.00)	Arson (1.00, 1.00)	1	[-0.748, 0.748]	0	1	0.00%
**Assault (54.1, 7.18)**	**Assault (39.4, 7.14)**	4.90×10^{-6}	[9.38, 20.1]	5.64	7.16	-27.2%
**Battery (131, 13.8)**	**Battery (100, 15.2)**	2.78×10^{-6}	[20.1, 41.8]	5.84	14.5	-23.7%
**Burglary (31.8, 6.67)**	**Burglary (16.5, 3.52)**	1.53×10^{-8}	[11.3, 19.3]	7.84	5.33	-48.1%
**Criminal Damage (79.7, 10.5)**	**Criminal Damage (56.5, 11.5)**	3.25×10^{-6}	[15.0, 31.5]	5.79	11.0	-29.1%
Criminal Sexual Assault (2.93, 2.19)	Criminal Sexual Assault (1.80, 1.08)	0.083	[-0.157, 2.42]	1.80	1.73	-38.6%
**Criminal Trespass (18.9, 5.81)**	**Criminal Trespass (8.73, 2.05)**	6.13×10^{-7}	[6.94, 13.5]	6.41	4.36	-53.8%
Homicide (1.67, 2.29)	Homicide (0.733, 0.704)	0.142	[-0.333, 2.20]	1.51	1.69	-56.1%
**Interference with Public Officer (2.80, 1.61)**	**Interference with Public Officer (0.267, 0.458)**	2.72×10^{-6}	[1.65, 3.42]	5.85	1.19	-90.5%
**Narcotics (35.0, 6.75)**	**Narcotics (5.00, 2.56)**	1.12×10^{-15}	[26.2, 33.8]	16.1	5.12	-85.7%
**Other Offense (50.4, 9.12)**	**Other Offense (24.8, 4.54)**	1.76×10^{-10}	[20.2, 31,0]	9.73	7.21	-50.8%
**Public Peace Violation (4.20, 1.37)**	**Public Peace Violation (1.47, 0.916)**	6.05×10^{-7}	[1.86, 3.61]	6.41	1.17	-65.0%
**Robbery (29.1, 5.34)**	**Robbery (15.8, 5.28)**	1.96×10^{-7}	[9.30, 17.2]	6.84	5.31	-45.7%
Sex Offense (2.67, 2.35)	Sex Offense (2.07, 1.49)	0.410	[-0.871, 2.07]	0.836	1.97	-22.5%
**Theft (156, 16.7)**	**Theft (82.0, 11.0)**	1.90×10^{-14}	[63.6, 84.8]	14.4	14.1	-47.4%
Weapons Violation (10.9, 3.73)	Weapons Violation (14.3, 4/42)	0.034	[-6.39, -0.276]	-2.23	4.09	31.2%

Table S14. Comparisons of 2017 crime types and 2020 crime types after the stay at home order was put in place. The Bonferroni correction with $n = 18$ produces a corrected significance value of $\alpha = 0.0027$ and the degrees of freedom are 28. There are a number of crime types that show significant differences between the two time periods.

2019 Crime Types - State of Emergency	2020 Crime Types - State of Emergency	p-value	ci	t-score	STDev	Percent Change
**Total Crimes (666, 50.6)**	**Total Crimes (538, 59.2)**	9.85×10^{-6}	[81.5, 175]	5.70	55.1	-19.2%
Gun Crimes (35.1, 5.97)	Gun Crimes (34.6, 7.60)	0.836	[-5.20, 6.37]	0.209	6.83	-1.42%
Arson (1.08, 1.08)	Arson (0.750, 1.06)	0.453	[-0.572, 1.24]	0.763	1.07	-30.6%
Assault (58.4, 8.71)	Assault (49.3, 7.39)	0.011	[2.33, 16.0]	2.78	8.08	-15.6%
Battery (135, 24.4)	Battery (120, 17.9)	0.101	[-3.17, 33.0]	1.71	21.4	-11.1%
Burglary (21.9, 5.99)	Burglary (18.5, 4.50)	0.129	[-1.07, 7.90]	1.58	5.30	-15.5%
Criminal Damage (69.5, 5.70)	Criminal Damage (60.0, 10.6)	0.012	[2.30, 16.7]	2.74	8.50	-13.7%
Criminal Sexual Assault (3.75, 2.01)	Criminal Sexual Assault (2.83, 2.04)	0.279	[-0.795, 2.63]	1.11	2.02	-24.5%
Criminal Trespass (18.1, 3.87)	Criminal Trespass (13.9, 5.21)	0.037	[0.279, 8.05]	2.22	4.59	-23.2%
Homicide (0.917, 0.900)	Homicide (0.667, 0.651)	0.444	[-0.415, -0.915]	0.779	0.786	-27.3%
Interference with Public Officer (3.58, 1.38)	Interference with Public Officer (3.00, 1.91)	0.400	[-0.826, 1.99]	0.859	1.66	-16.2%
**Narcotics (42.8, 6.75)**	**Narcotics (28.3, 10.3)**	4.83×10^{-4}	[7.15, 21.9]	4.09	8.68	-33.9%
Other Offense (45.8, 6.52)	Other Offense (36.0, 8.49)	0.004	[3.43, 16.24]	3.18	7.57	-21.4%
Public Peace Violation (5.08, 2.31)	Public Peace Violation (2.67, 2.53)	0.023	[0.362, 4.47]	2.44	2.43	-47.4%
Robbery (15.6, 3.50)	Robbery (16.7, 5.37)	0.564	[-4.92, 2.75]	-0.586	4.53	7.05%
Sex Offense (3.33, 2.31)	Sex Offense (2.83, 2.04)	0.580	[-1.34, 2.34]	0.562	2.18	-15.0%
**Theft (144, 17.0)**	**Theft (113, 20.5)**	5.15×10^{-4}	[15.3, 47.2]	4.06	18.8	-21.5%
Weapons Violation (15.6, 3.87)	Weapons Violation (14.4, 3.70)	0.459	[-2.04, 4.37]	0.754	3.79	-7.69%

Table S15. Comparisons of 2019 crime types after the state of emergency was announced, but before the stay at home order was put in place. The Bonferroni correction with $n = 18$ produces a corrected significance value of $\alpha = 0.0027$ and the degrees of freedom are 22. There are a number of crime types that show significant differences between the two time periods.

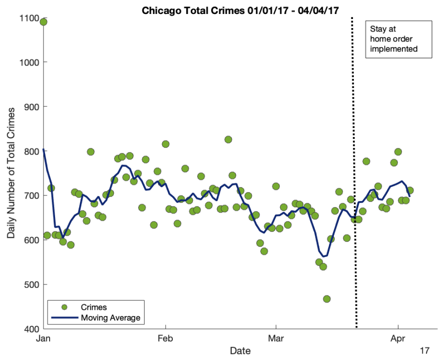

(a) 2017

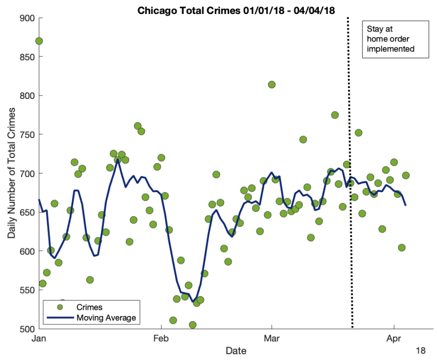

(b) 2018

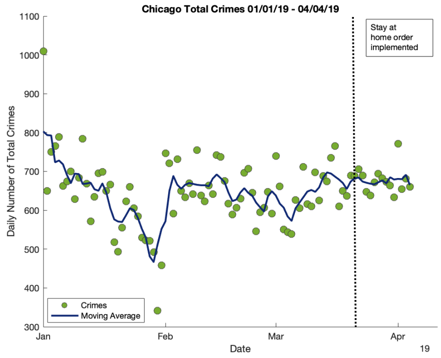

(c) 2019

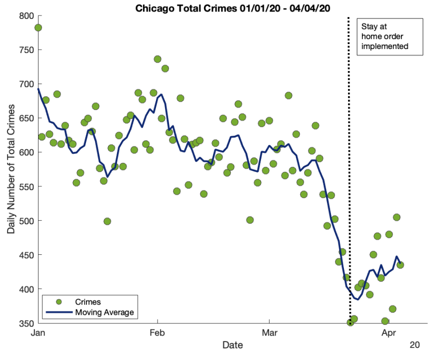

(d) 2020

Fig S13. The total crime trends seen in Chicago from 2017-2020 over a three month time period spanning from January to the end of March. Each point represents the total number of crimes which occurred on that day. The vertical line shows when the stay at home order was implemented in 2020 (3/21/20) for ease of comparison between the three years. The navy line represents the moving average of the data. The years 2017, 2018, and 2019 show fairly consistent variation in total crimes, while 2020 shows a sharp decline after implementation of the stay-at-home order.

2018 Crime Types - State of Emergency	2020 Crime Types - State of Emergency	p-value	ci	t-score	STDev	Percent Change
**Total Crimes (686, 43.8)**	**Total Crimes (538, 59.2)**	5.89×10^{-7}	[103, 191]	6.93	55.1	-21.6%
Gun Crimes (36.9, 6.93)	Gun Crimes (34.6, 7.60)	0.440	[-3.82, 8.49]	0.786	7.27	-6.23%
Arson (0.667, 0.779)	Arson (0.750, 1.06)	0.828	[-0.868, 0.702]	-0.220	0.927	12.4%
Assault (52.3, 9.67)	Assault (49.3, 7.39)	0.390	[-4.20, 10.4]	0.878	8.60	-5.74%
Battery (135, 21.0)	Battery (120, 17.9)	0.072	[-1.44, 31.6]	1.89	19.5	-11.1%
**Burglary (26.8, 5.80)**	**Burglary (18.5, 4.50)**	7.85×10^{-4}	[3.85, 12.65]	3.89	5.19	-31.0%
Criminal Damage (74.9, 13.5)	Criminal Damage (60.0, 10.6)	0.006	[4.67, 25.2]	3.02	12.1	-19.9%
Criminal Sexual Assault (3.75, 2.73)	Criminal Sexual Assault (2.83, 2.04)	0.362	[-1.12, 2.96]	0.931	2.41	-24.5%
Criminal Trespass (18.3, 2.53)	Criminal Trespass (13.9, 5.21)	0.017	[0.865, 7.80]	2.59	4.10	-24.0%
Homicide (1.17, 1.19)	Homicide (0.667, 0.651)	0.216	[-0.314, 1.31]	1.27	0.961	-43.0%
Interference with Public Officer (3.33, 1.37)	Interference with Public Officer (3.00, 1.91)	0.628	[-1.07, 1.74]	0.492	1.66	-9.91%
**Narcotics (40.8, 5.62)**	**Narcotics (28.3, 10.3)**	0.001	[5.50, 19.5]	3.70	8.27	-30.6%
Other Offense (45.1, 8.10)	Other Offense (36.0, 8.49)	0.014	[2.06, 16.1]	2.68	8.29	-20.2%
Public Peace Violation (3.92, 2.43)	Public Peace Violation (2.67, 2.53)	0.231	[-0.852, 3.35]	1.23	2.48	-31.9%
**Robbery (25.6, 6.92)**	**Robbery (16.7, 5.37)**	0.002	[3.67, 14.2]	3.53	6.19	-34.8%
Sex Offense (2.75, 1.71)	Sex Offense (2.83, 2.04)	0.915	[-1.68, 1.51]	-0.109	1.88	2.91%
**Theft (153, 11.6)**	**Theft (113, 20.5)**	8.14×10^{-6}	[25.2, 53.4]	5.78	16.7	-26.1%
Weapons Violation (13.1, 3.34)	Weapons Violation (14.4, 3.70)	0.365	[-4.32, 1.65]	-0.926	3.53	9.92%

Table S16. Comparisons of 2018 crime types after the state of emergency was announced, but before the stay at home order was put in place. The Bonferroni correction with $n = 18$ produces a corrected significance value of $\alpha = 0.0027$ and the degrees of freedom are 22. There are a number of crime types that show significant differences between the two time periods.

2017 Crime Types - State of Emergency	2020 Crime Types - State of Emergency	p-value	ci	t-score	STDev	Percent Change
Total Crimes (624, 73.2)	Total Crimes (538, 59.2)	0.005	[29.7, 142]	3.17	66.6	-13.8%
Gun Crimes (31.8, 8.83)	Gun Crimes (34.6, 7.60)	0.509	[-9.81, 4.14]	-0.842	8.24	8.81%
Arson (0.917, 0.996)	Arson (0.750, 1.06)	0.695	[-0.702, 1.04]	0.398	1.03	-18.2%
Assault (43.8, 7.94)	Assault (49.3, 7.39)	0.093	[-12.0, 0.995]	-1.76	7.67	12.6%
Battery (121, 19.6)	Battery (120, 17.9)	0.872	[-14.6, 17.1]	0.163	18.8	-0.826%
**Burglary (29.2, 4.73)**	**Burglary (18.5, 4.50)**	1.08×10^{-5}	[6.76, 14.6]	5.66	4.62	-36.6%
Criminal Damage (70.2, 18.0)	Criminal Damage (60.0, 10.6)	0.106	[-2.34, 22.7]	1.69	14.8	-14.5%
Criminal Sexual Assault (3.33, 1.67)	Criminal Sexual Assault (2.83, 2.04)	0.518	[-1.08, 2.08]	0.658	1.86	-15.0%
Criminal Trespass (17.3, 6.27)	Criminal Trespass (13.9, 5.21)	0.161	[-1.47, 8.30]	1.45	5.77	-19.7%
Homicide (1.50, 1.31)	Homicide (0.667, 0.651)	0.062	[-0.045, 1.71]	1.97	1.04	-55.5%
Interference with Public Officer (2.25, 1.29)	Interference with Public Officer (3.00, 1.91)	0.271	[-2.13, 0.628]	-1.13	1.63	33.3%
Narcotics (33.8, 8.45)	Narcotics (28.3, 10.3)	0.172	[-2.54, 13.4]	1.41	9.40	-16.3%
**Other Offense (48.0, 5.86)**	**Other Offense (36.0, 8.49)**	5.60×10^{-4}	[5.83, 18.2]	4.03	7.29	-25.0%
Public Peace Violation (4.25, 2.63)	Public Peace Violation (2.67, 2.53)	0.148	[-0.605, 3.77]	1.50	2.58	-37.2%
Robbery (24.6, 7.00)	Robbery (16.7, 5.37)	0.005	[2.64, 13.2]	3.11	6.24	-32.1%
Sex Offense (2.75, 1.29)	Sex Offense (2.83, 2.04)	0.906	[-1.53, 1.36]	-0.120	1.70	2.91%
Theft (133, 21.9)	Theft (113, 20.5)	0.036	[1.37, 37.3]	2.23	21.2	-15.0%
**Weapons Violation (8.67, 2.81)**	**Weapons Violation (14.4, 3.70)**	3.00×10^{-4}	[-8.53, -2.97]	-4.29	3.29	66.1%

Table S17. Comparisons of 2017 crime types after the state of emergency was announced, but before the stay at home order was put in place. The Bonferroni correction with $n = 18$ produces a corrected significance value of $\alpha = 0.0027$ and the degrees of freedom are 22. There are a number of crime types that show significant differences between the two time periods.

Crime Type Comparisons For 2020, our main year of interest, in the main paper text we presented scatter plots of a few relevant crime types that are significantly different between the stay at home period and the time period before COVID. In S14 Fig we present scatter plots of the other crime types showing significant differences:

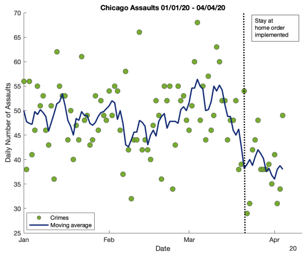

(a) Assaults

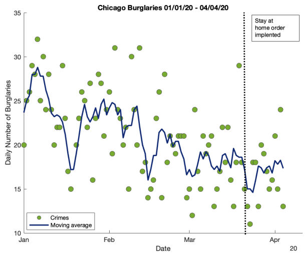

(b) Burglaries

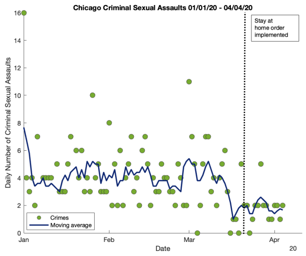

(c) Criminal Sexual Assault

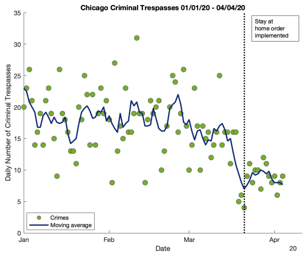

(d) Criminal Trespasses

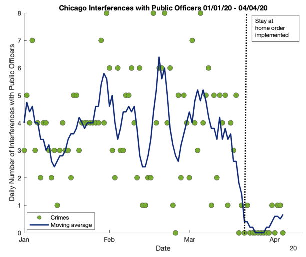

(e) Interferences with Public Officers

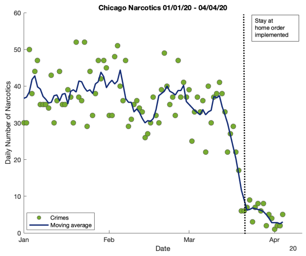

(f) Narcotics

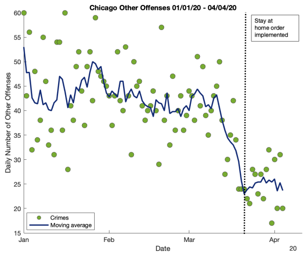

(g) Other Offenses

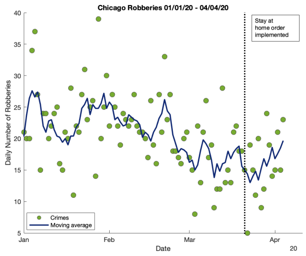

(h) Robberies

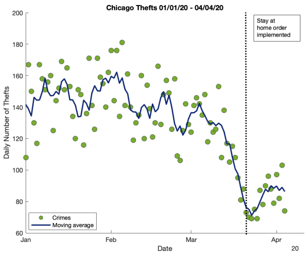

(i) Thefts

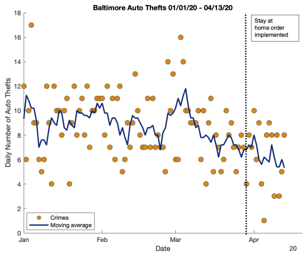

(j) Auto Thefts

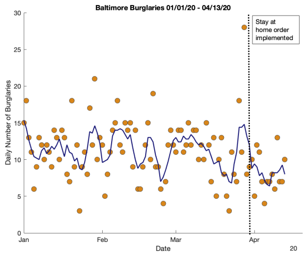

(k) Burglaries

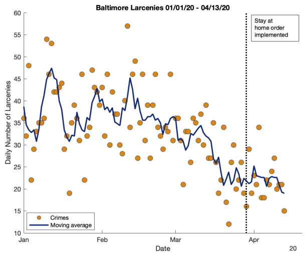

(l) Larcenies

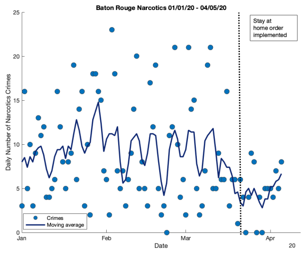

(m) Narcotics

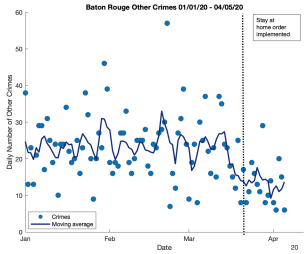

(n) Other Crimes

Fig S14. Crime types across the three cities that showed significant differences during the time period before stay at home orders were put in place and after they were implemented in 2020. Each dot represents the total number of that crime type which occurred daily. The vertical line represents when the stay at home order was put in place for the city. The navy line represents the moving average of the data. All of the crime types show declines after implementation of the stay-at-home order.

Time Period Comparisons for Baltimore and Baton Rouge

We were also curious as to whether comparing the early 2020 dataset (January - early April) to the time period before stay at home orders were implemented in Baltimore and Baton Rouge would produce significant differences (as in Table S3 and S6). As shown in Tables S18 and S19, there are no significant differences in either city, further confirming that the first two weeks of the stay at home order time period are correlated with differences in crime dynamics.

Early 2020 Crime Types	Pre-SAH 2020 Crime Types	p-value	ci	t-score	STDev	Percent Change
Total Crimes (98.7, 17.9)	Total Crimes (103, 15.4)	0.085	[-9.01, 0.591]	-1.73	16.8	4.36%
Gun Crimes (12.6, 6.60)	Gun Crimes (12.8, 6.70)	0.784	[-2.16, 1.64]	-0.274	6.65	1.59%
Assault (32.4, 7.85)	Assault (33.2, 7.84)	0.474	[-3.06, 1.43]	-0.717	7.85	2.47%
Arson (0.202, 0.427)	Arson (0.216, 0.441)	0.824	[-0.138, 0.110]	-0.223	0.433	6.93%
Auto Theft (8.46, 2.81)	Auto Theft (8.85, 2.68)	0.328	[-1.18, 0.396]	-0.980	2.75	4.61%
Burglary (10.9, 4.04)	Burglary (11.4, 4.07)	0.395	[-1.66, 0.658]	-0.853	4.05	4.59%
Homicide (0.779, 1.09)	Homicide (0.761, 1.13)	0.914	[-0.300, 0.335]	0.109	1.11	-2.31%
Larceny (32.4, 9.48)	Larceny (34.3, 8.83)	0.149	[-4.55, 0.698]	-1.45	9.19	5.86%
Rape (0.481, 0.737)	Rape (0.511, 0.773)	0.780	[-0.246, 0.185]	-0.280	0.754	6.24%
Robbery (11.5, 4.51)	Robbery (12.1, 4.30)	0.387	[-1.82, 0.707]	-0.867	4.41	5.22%
Shooting (1.48, 1.63)	Shooting (1.49, 1.68)	0.974	[-0.481, 0.465]	-0.033	1.65	0.676%

Table S18. Comparisons of Baltimore's early 2020 dataset to the time period before the stay-at-home order was put in place. The stay at home order was put in place on 03/30/20. The Bonferroni correction uses $n = 11$, giving an adjusted significance value of $\alpha = 0.00455$ and the degrees of freedom are 190. There are no crime types that show significant differences between these two time periods.

Early 2020 Crime Types	Pre-SAH 2020 Crime Types	p-value	ci	t-score	STDev	Percent Change
Total Crimes (109, 21.3)	Total Crimes (113, 20.0)	0.188	[-10.3, 2.05]	-1.32	20.7	3.67%
Assault (6.28, 2.88)	Assault (6.45, 3.03)	0.706	[-1.05, 0.711]	-0.379	2.95	2.71%
Battery (9.41, 4.20)	Battery (9.84, 4.38)	0.507	[-1.71, 0.849]	-0.665	4.29	4.57%
Burglary (13.4, 5.55)	Burglary (13.7, 5.75)	0.739	[-1.97, 1.40]	-0.334	5.64	2.24%
Criminal Damage (9.22, 3.63)	Criminal Damage (9.03, 3.59)	0.723	[-0.885, 1.27]	0.355	3.61	-2.06%
Firearm (5.11, 3.63)	Firearm (5.21, 3.82)	0.862	[-1.21, 1.01]	-0.174	3.72	1.96%
Homicide (1.25, 1.19)	Homicide (1.20, 1.14)	0.785	[-0.312, 0.412]	0.273	1.21	-4.00%
Juvenile (1.08, 1.20)	Juvenile (1.13, 1.27)	0.824	[-0.410, 0.326]	-0.223	1.23	4.63%
Narcotics (8.46, 5.82)	Narcotics (9.23, 5.97)	0.391	[-2.53, 0.993]	-0.860	5.891	9.10%
Nuisance (1.71, 1.34)	Nuisance (1.78, 1.26)	0.736	[-0.456, 0.323]	-0.338	1.30	4.09%
Other (21.7, 9.33)	Other (23.4, 8.94)	0.220	[-4.44, 1.03]	-1.23	9.16	7.83%
Robbery (1.29, 1.26)	Robbery (1.31, 1.23)	0.912	[-0.392, 0.351]	-0.112	1.24	1.55%
Sexual Assault (0.490, 0.649)	Sexual Assault (0.513, 0.675)	0.819	[-0.220, 0.175]	-0.229	0.661	4.69%
Theft (28.5, 7.53)	Theft (29.3, 7.39)	0.491	[-3.01, 1.45]	-0.691	7.47	2.81%
Vice (0.573, 0.736)	Vice (0.575, 0.708)	0.985	[-0.218, 0.214]	-0.019	0.723	0.349%

Table S19. Comparisons of Baton Rouge's early 2020 dataset compared to the time before the stay at home order was put in place. The stay at home order was put in place on 03/22/20. The dataset has 174 degrees of freedom and the Bonferroni correction uses $n = 15$ and an adjusted significance value of $\alpha = 0.00333$. There are no crime types that show significant differences between these two time periods.
