# Supplementary material for: COVID-19 and crime: Analysis of crime dynamics amidst social distancing protocols
Source: PLoS One. 2021 Apr 1;16(4):e0249414. doi: 10.1371/journal.pone.0249414 (PMC8016314; doi:10.1371/journal.pone.0249414)
Supplement: Attachment — Submitted filename: PONE-D-20-31968.pdf [file pone.0249414.s002.pdf]

Dear Editor,

This is a report on the paper "COVID-19 and crime: Analysis of crime dynamics amidst social distancing protocols" submitted by S.M. Scott and L. J. Gross for publication in PLoS One. The paper deals with changes in crime in several US cities (Chicago, Baltimore, Baton Rouge) after the establishment of COVID-19 related lockdowns. In particular, the authors conclude that the stay at home orders contributed to decreasing crime rates in the three cities above, with property crimes declining more.

The topic is of course timely and interesting but paper feels like it was hastily written. It is lacking details, context, background, and often uniform approach among the cities.

For example, for Chicago they use 20 out of 32 types of crime for their statistics, with a cutoff of each crime being at least 0.2 percent of total tally. For the other cities, no criteria is presented. The crime lists don't include anything else than the names of the crimes (we don't get to learn about numbers, locations, definitions, which ones are considered felonies etc). In some case the nomenclature is bizarre - is "juvenile" a crime? How is "vice" defined? How are "gun crimes" different than "shooting"? What is the crime of "criminal damage"?

Similarly the t-Test section is poorly presented. We are not told what alpha is, what is n, how the Bonferroni (or is it Bonferonni? It is spelt in two different ways) correction is supposed to affect the t-Test. They speak of an experiment within the context of the t-Test, but it is not clear what experiment they are referring to. What is a "year in year" test? Later they call it a "pair-wise t-test appropriately corrected for replication" without any context of what the replication is.

Finally, in the results section they speak of "victim-based crime data from Baltimore", what exactly does this mean? Is it specific to Baltimore? Is crime from Chicago not victim-based?

They also discuss Baton Rouge as a "city with different demographics" what does this mean? Is it in terms of race? age? wealth? Up until this point (page 5) there has been no discussion on demographics of the different cities. Indeed, we are not even told what the population numbers are.

In Table 1 \sigma and \mu are not defined. Nor is the p-value.

In the Conclusions it is not clear what their message is. Consider these two statements made by the authors:

1) Chicago's total crimes during the first three months of the year (including the time period following the stay at home order) declined in 2020 compared to 2017, 2018, and 2019.

2) Before COVID protocols were put in place, there were no significant changes in total crimes compared to 2018 and 2019.

Since COVID-19 became a problem in March 2020, and all the protocols came after March 2020, what are we to believe? Did crime within January 2020-March 2020 decline (as the first sentence would imply!) or not (as the second sentence would imply)?

Then they add nuances that confuse the reader even more:

3) There were some changes in particular crime types that may have been due to social factors, policing protocols, or other mechanisms. There were also significant changes in total crimes between 2017 and 2020, with all but weapons violations showing declines.

So, did all these crimes except for weapons violations decrease? Also what does it mean that there were changes in "crime types?" Did the definition change? This is unclear. They also discuss interpersonal crimes, but do not define an interpersonal crime. Is robbery an interpersonal crime? Unclear.

In conclusion, in its present format the paper is not suitable for publication. The authors need to do a thorough re-write and make sure everything is defined and explained in a pedagogical manner. The topic as mentioned is timely and important, but the presentation to be clearer and unambiguous.
