# Supplementary material for: COVID-19 and crime: Analysis of crime dynamics amidst social distancing protocols
Source: PLoS One. 2021 Apr 1;16(4):e0249414. doi: 10.1371/journal.pone.0249414 (PMC8016314; doi:10.1371/journal.pone.0249414)
Supplement: Attachment — Submitted filename: Response to Reviewers.pdf [file pone.0249414.s003.pdf]

THE UNIVERSITY OF TENNESSEE KNOXVILLE

Response to Reviewers

24 December 2020

Dear Dr. Topaz,

First, we would like to thank you and the two anonymous reviewers for the comments and for the helpful feedback provided, which has enhanced the manuscript in many ways. We greatly appreciate the effort that the reviewers made to constructively provide advice about the manuscript and believe the resulting aper is much stronger due to their reviews. Below is our response to the comments provided on our manuscript entitled, "COVID-19 and crime: Analysis of crime dynamics amidst social distancing protocols." Our responses follow each comment in italics. Actions that have been taken or sections that have been added are colored in green.

Reviewer #1 Comments

1) I don't understand the choice to only analyze two weeks following the stay-at-home orders. I think the paper would be significantly strengthened by including more data after the stay-at-home orders. We are now well past the end date of those orders, so it makes sense to check and see if crime indeed went back up (or didn't).
We chose to analyze the two weeks following the implementation of stay-at-home orders due to the likelihood that stronger adherence to stay at home orders would occur in the initial time period following the announcement (see figures S1-S3). From the data, all three metrics showed the best adherence to social distancing protocols immediately following their implementation. The crime data from 2020 (see figures S4-S6) also show that April was the month with the lowest number of crimes. Therefore, determining whether there is a correlation between social distancing protocols and crime will be most clear when tested in this time period. We are also aware that the full time period should be tested now that more data are available but wanted to highlight the initial impacts of these protocols. We have added more explanation within the manuscript as to our decision to test these two weeks, which is further supported by new figures within the supplemental information.

2) I feel like the authors were very thorough in comparing changes in the 2020 data to previous years to make sure the effects were not due to seasonality for Chicago, but why not for the other two cities? Is it fair to only use the 2020 data for those two cities after performing such an in-depth analysis of Chicago to claim that the changes in their crimes were in fact due the stay-at-home order?
Chicago is often mentioned as a major hub for gun crime in the United States, making it an important study system for crime in general. Also, it was one of the major cities in the United States which experienced an early COVID-19 outbreak. The initial purpose of this study was

Department of Ecology and Evolutionary Biology
569 Dabney Hall, Knoxville, TN 37996-1610
865-974-3065 865-974-3067 fax

to observe changes that occurred in Chicago following the implementation of stay-at-home orders, but we then determined that it would be helpful to determine whether these patterns held across other cities. To address this in the manuscript, we have altered our paper objectives to state that Chicago is our main study area and that the comparisons with Baltimore and Baton Rouge were to support our conclusions about Chicago. We have also added line plots highlighting the temporal changes in Baltimore and Baton Rouge over the last three years to allay concerns that differences in the stay-at-home time period were due to seasonality. In the supplementary material, the line plots added also include monthly total crime data for 2020 through October as a basis for potential future discussions of longer-term impacts of behavior on total crime.

3) Why scatter plots? They are super hard to read. For the t-test, aren't you just taking total number before and after? Why not show just that? Or a moving average if you want to show time effects?
We chose to use scatter plots in order to highlight the variation present in the datasets and to highlight the differences in pre- vs. post-stay-at-home order crime. We have improved the clarity of the scatter plots and added in a 5-day moving average trend line and discussed the calculation of this in the supplementary material. This moving average analysis did not add any new conclusions but may improve clarity of the underlying trends for readers. In terms of the t-tests, we have further clarified the objectives of the t-tests to compare the average number of crimes per day pre- and post-stay-at-home orders (as well as for comparisons of time periods from previous years over the same seasons).

4) What were the stay-at-home orders in the different cities? Did they all have similar stay-at-home orders Similar punishments for breaking the order? Any way to gauge how well people actually listened?
The stay-at-home orders were generally the same across the three cities. They closed all non-essential businesses, mandated wearing masks, and encouraged citizens to minimize non-essential visits outside their homes. The punishments for stay-at-home orders included fines or potential jail time in Maryland and Illinois, but Louisiana did not enforce fines. Information regarding the mandates and adherence to stay-at-home orders have been added to the manuscript. We note that in the three metrics for adherence (change in distance traveled, non-essential visits, and encounter density) across all three cities, the lowest values over the year (through November) occurred during the initial time period following implementation of stay-at-home-orders (see figures S1-S3). We have added figures highlighting adherence to the supplemental information.

5) The introduction seems to concentrate on the idea of interpersonal vs. property crimes, but there doesn't seem to be much discussion or analysis of this in the text. Perhaps a visualization here would be nice where you color-code crimes that are considered personal and property and show in which city they increased, decreased or stayed the same.
This is a great suggestion. The crime types for each city are now broken down into interpersonal vs. property vs. statutory crimes using (1, 2, and 3) to improve understanding within the manuscript. The specific crime types are defined in the supplementary material (table S2). We created a bar chart of the different crime types pre- and post-stay-at-home orders that is now in the supplementary material (see figures S7-S12) to show how crime categories shifted over these time periods. We have also added a statement regarding the

lack of uniformity of crime data availability across the U.S., which constrains the ability to match exact crime types between cities.

6) All of the tables in the main text and the SI are too hard to digest. I think summarizing the results in figures in the main paper and referencing those tables might be better.
We have constructed bar charts showing the differences in the three crime categories between time periods (see figures S7-S12). We have also added the percent and direction change to the tables in order to clarify the importance of each crime type and category's contributions to the overall decline in total crimes across the three cities.

7) In general, I feel that this paper would be much easier to understand if the authors put some thought into creating figures that summarize the data and support their conclusions. Here are some suggestions:
   a. For Figure 1, plot the total crime in first three months of 2020 compared to 2019 and 2018 and compute the percent change.
   This is a great suggestion. The percent change has been included for each table (see tables 1 and 2, S3-S19).

   b. For Figure 2, color code the crimes as property or interpersonal and plot the change in number of crimes for each city that were statistically significant. I'd like to know from this graph, which crimes had similar behavior across the three cities and by how much did they change.
   We have created bar charts for changes pre- and post-stay-at-home-order for the three crime categories in each of the three cities to allow comparisons across cities (see figures S7-S12). Due to the lack of uniformity in publicly available data, comparisons of exact crime types between cities are difficult (e.g., theft data in Chicago differs from auto theft data in Baltimore), but we hope the inclusion of crime categories and percent change within the table clarifies the patterns observed in the data.

8) In the SI text, a comparison of the total number (or average number) of crimes between same time periods of 2019 compared to 2020 (summarizing Tables S1 - S7) would be good. You can even include this in the first figure summarizing Table 1 by breaking down the first three months to the three time periods that are analyzed in the SI text. Again, I wouldn't use scatter plots to visualize the data since the actual statistical analysis that's being performed is simply on the total number of crimes before and after a date.
The purpose of a t-test is to compare the means across the two time periods. The data show the day-to-day variability in occurrence of each crime. Scatter plots allow this variability to be demonstrated for readers to encourage appreciation of this variability in cases for which there are significant differences seen across the two time periods. We have added line graphs showing the monthly total crime dynamics across the three cities in each year 2017-2019 and January-October of 2020 (see figures S4-S6). We have also shown in tables S3-S19 the percent change to further clarify the dynamics between the time periods of interest.

Smaller concerns:

9) The introduction is lacking a bit and I would appreciate a bit more motivation for choosing these three particular crimes sets, what they have in common, and what you found (be specific, by how much did crime overall seem to change during the stay-at-home order?).

We have added more information in the introduction to clarify the motivation and conclusions of this study for both reviewers.

10) Write out less than or equal to rather than using the math symbol within paragraphs (e.g., lines 68, 123, 124).
   This has been updated.

11) Additional and in line 133
   This change has been made.

12) Say what sigma and mu are in Table 1 and 2 captions.
   Mu and sigma have been defined in the table captions.

13) A bit more explanation on the Bonferroni correction and what you are using in the t-tests might be nice for readers that don't have a background in statistics.
   Further explanation of t-tests and the Bonferroni correction have been added to the manuscript.

<u>Reviewer #2 Comments</u>

1) For example, for Chicago they use 20 out of 32 types of crime for their statistics, with a cutoff of each crime being at least 0.2 percent of total tally. For the other cities, no criteria are presented. The crime lists don't include anything else than the names of the crimes (we don't get to learn about numbers, locations, definitions, which ones are considered felonies etc). In some case the nomenclature is bizarre - is "juvenile" a crime? How is "vice" defined? How are "gun crimes" different than "shooting"? What is the crime of "criminal damage"?
   For Baton Rouge and Baltimore, all crime types available have been included. Meanwhile, the Chicago dataset has far more crime types available. Some of them only have one or two occurrences throughout the entire year and no occurrences during the time periods we were observing in this study. To determine the appropriate percentage of total crime cutoff, we found the crime type in Baltimore or Baton Rouge with the lowest percentage of total crimes and used this as the baseline to determine our cutoff for Chicago. We have highlighted this explanation in the manuscript for clarity. Each dataset includes different information since there is no consensus on how to collect, define, and present publicly available crime data. We have added a statement about data quality to the manuscript and included definitions for each crime type in the supplemental information (see table S2).

2) Similarly, the t-Test section is poorly presented. We are not told what alpha is, what is n, how the Bonferroni (or is it Bonferonni? It is spelt in two different ways) correction is supposed to affect the t-Test. They speak of an experiment within the context of the t-Test, but it is not clear what experiment they are referring to. What is a "year in year" test? Later they call it a "pair-wise t-test appropriately corrected for replication" without any context of what the replication is.
   The t-test is a comparison of means from two different populations (in this case, different time periods), in order to determine whether the data come from the same distribution. Alpha is the level of significance desired, n is the total size of our sample, and the Bonferroni correction keeps random results from showing significance due to the number of t-tests we are using. When a researcher completes a large volume of any sort of statistical test, there is

a random chance some of them will come up as significant due to repetition. The Bonferroni correction helps to tighten the restrictions on what is significantly different in order to prevent these errors due to replication. We have added further descriptions and definitions to the manuscript in order to clarify the statistical methods used and the comparisons undertaken. We have also corrected the spelling of Bonferroni within the manuscript.

3) Finally, in the results section they speak of "victim-based crime data from Baltimore", what exactly does this mean? Is it specific to Baltimore? Is crime from Chicago not victim-based?
The crime data available from Baltimore is narrowed to only crimes in which there are victims. From the dataset, victim-based crime is defined as crimes in which someone is victimized (either personally or in terms of property). Meanwhile, Chicago and Baton Rouge have crime datasets that are comprised of more crime types. We have added clarifications into the materials and methods about what each of these crime datasets include. We have also divided the crime types into three different crime categories (property, statutory, and interpersonal). These crime categories are highlighted in the tables (1 and 2), as well as being used to create bar graphs that show shifts between pre- and post-stay-at-home order time periods (see figures S7-S12). The supplemental information now also includes descriptions of each crime type used from the datasets (see table S2).

4) They also discuss Baton Rouge as a "city with different demographics" what does this mean? Is it in terms of race? age? wealth? Up until this point (page 5) there has been no discussion on demographics of the different cities. Indeed, we are not even told what the population numbers are.
Baton Rouge, Chicago, and Baltimore are all cities regularly studied for their crime dynamics. Chicago is the largest city, followed by Baltimore and then Baton Rouge. Each of them has differing poverty levels, but all fall above the national average (10.5%). Chicago is the densest, followed by Baltimore and then Baton Rouge. Therefore, these cities have similarities, but also differences that make them interesting comparisons. We have added more information about the cities themselves into the descriptions of the materials and methods to address this. We have also added a table of demographic information to the supplemental material for further clarification (see table S1).

5) In Table 1 \sigma and \mu are not defined. Nor is the p-value.
All of these terms have been defined in the manuscript.

6) In the Conclusions it is not clear what their message is. Consider these two statements made by the authors:
1) Chicago's total crimes during the first three months of the year (including the time period following the stay-at-home order) declined in 2020 compared to 2017, 2018, and 2019.
2) Before COVID protocols were put in place, there were no significant changes in total crimes compared to 2018 and 2019.
Since COVID-19 became a problem in March 2020, and all the protocols came after March 2020, what are we to believe? Did crime within January 2020-March 2020 decline (as the first sentence would imply!) or not (as the second sentence would imply)?
Then they add nuances that confuse the reader even more:

3) There were some changes in particular crime types that may have been due to social factors, policing protocols, or other mechanisms. There were also significant changes in total crimes between 2017 and 2020, with all but weapons violations showing declines.
So, did all these crimes except for weapons violations decrease? Also, what does it mean that there were changes in "crime types?" Did the definition change? This is unclear. They also discuss interpersonal crimes, but do not define an interpersonal crime. Is robbery an interpersonal crime? Unclear.
The major conclusion of this paper is that there were significant changes that occurred in the two weeks after the stay-at-home orders were put in place across all three cities and that more work needs to be done to analyze the impacts that changes in social dynamics have on crime. This conclusion was supported by first making sure that 2020 was not showing unexpected crime dynamics in Chicago before the pandemic began. We then checked to be sure that there were not seasonal dynamics in place by comparing time periods of past years to the crime dynamics in 2020 (see figures S4, S13). Once we were certain that, generally, there are no existing temporal patterns at play, we then test to see whether the stay-at-home orders had a significant impact on the crime dynamics in Chicago and then, for comparison, two other cities. We have clarified the conclusions of the manuscript and added more figures to the supplementary material for ease of reader understanding. Specifically, we have added descriptions of crime types and data quality, divided the crime types into crime categories, and carried these definitions throughout the manuscript.

Sincerely,

Shelby M. Scott
National Defense Science and Engineering Graduate Fellow
Department of Ecology and Evolutionary Biology

Louis J. Gross
Chancellor's Professor and Alvin and Sally Beaman Distinguished Professor of Ecology and Evolutionary Biology and Mathematics
Director, National Institute for Mathematical and Biological Synthesis
Director, The Institute for Environmental Modeling, University of Tennessee
Past-President, The Society for Mathematical Biology
