# Supplementary material for: COVID-19 and crime: Analysis of crime dynamics amidst social distancing protocols
Source: PLoS One. 2021 Apr 1;16(4):e0249414. doi: 10.1371/journal.pone.0249414 (PMC8016314; doi:10.1371/journal.pone.0249414)
Supplement: Attachment — Submitted filename: Reponse to Reviewers.pdf [file pone.0249414.s004.pdf]

THE UNIVERSITY OF TENNESSEE KNOXVILLE

Response to Reviewers

9 March 2021

To whom it may concern,

First, we would like to thank the anonymous reviewer for their service and for the helpful feedback provided on our revision, which has enhanced the manuscript in many ways. Below is our response to the comments provided on our revision of the manuscript entitled, "COVID-19 and crime: Analysis of crime dynamics amidst social distancing protocols." Our responses follow each comment in italics. Actions that have been taken or sections that have been added are colored in green.

**Major points**

1. The introduction should clearly state the main conclusion of the paper. It currently states "changes in crime dynamics in all three cities .. are not uniform .. there are differences" What are those differences? Being clear about which city had significant changes in which type of crime and by how much up front will make the rest of the paper easier to digest.
*This is helpful feedback. We have explicitly listed the percent and direction change for each city's overall crime, each of the crime types that significantly changed, and their percent and direction of change. The rest of the paper then outlines the support for these claims.*

2. Some ways to better support the hypothesis in the text:

a. Label the crime types using P, S, and I rather than (1), (2), and (3) so it is easier for the reader to understand
*We have added letter rather than number designations for crime categories throughout the manuscript.*
b. Rearrange Table 1 and Table 2 to group crimes of a similar type rather than alphabetically. This way, the reader can easily see that the greatest change in crime was in P and S types, not I. Similarly, for the list of crimes in each city in the Data section.
*We have rearranged the tables and lists within the manuscript to reflect this change.*
c. Re-think Figure 2. The purpose of this figure is unclear to me. It is referenced in the text (e.g., in line 249) as supporting the hypothesis that P and S type crimes significantly decreased during SAH orders. This figure, however, doesn't clearly show that. Instead, it seems to show only two crimes that significantly decreased during this time period for each city. It doesn't even state which category those crimes are in. I wonder if it would be more useful to show ALL crimes that had significant decreased in each city and again order them by crime type (or color code by crime type, not city) to see that they are mostly type S or P. Figure 2a, b is not referenced in the text.

Department of Ecology and Evolutionary Biology
569 Dabney Hall, Knoxville, TN 37996-1610
865-974-3065 865-974-3067 fax

*Thank you for this incredibly helpful feedback. We have created a new Figure 2 that shows all of the crime types for the three cities, grouped by crime category, comparing average daily crime pre- and post-stay-at-home order implementation. The significant crime types are denoted with an asterisk next to the label. The past figure has been moved into the supplemental information (Fig S14) and now shows the time series with moving average trendline for all of the significant crime types in the three cities.*

d. Along the same lines as the point above, Fig S10-S12 does attempt to demonstrate this point (and is referenced in line 249) but is lacking. I would include all crime types for each city (also label them by type instead of number or remind the reader in the caption what the number represents) and clearly label which exhibit significant decreases (this is typically done with an asterisk above the two bars). It might also be clearer to color code by crime type, not city, since the main point is that all three cities demonstrated the greatest change in similar crime types.
*We have added a new Figure 2 that includes all the available crime types, grouped by crime category and have indicated which show significant different using an asterisk. We have maintained the color schemes based on cities for continuity between the main text and supplemental information but have split the crime types into crime categories throughout the main text for ease of understanding.*

**Other points**

1. The t-test indicates that there was a significant change in the number of crimes before and after the SAH order was implemented, but the scatter plots show a decrease in crime count prior to this date. This point should be addressed in limitations. The type of analysis that was performed cannot determine the date at which the crime changed. For example, if you chose to perform the same test before and after 3/1 instead, it looks from the scatter data that there would still be a significant decrease in total crimes in Chicago (Fig 1a).
*This is a great point, and we agree that there was a visual decline prior to implementation of the stay-at-home order. In the supplementary information (Table S7), we show that total crimes did decline in the state-of-emergency time period between the pre-COVID and stay-at-home order windows, but that this decline was not significant. We have added this information to the manuscript and also added to our limitations that we cannot know the exact date of the change in dynamics.*

2. It's unclear what is considered as the time period before SAH (e.g., in caption of Table 2 there are dates for the SAH and the two weeks after. What are the dates for before? Jan 1 - 3/21?). This should be clearly stated.
*The time period before spans from the beginning of the year 1/1/20 until before the stay-at-home order was implemented on 3/21/20. We have clarified this in the methods section of the manuscript.*

3. The SI text needs a bit more information to be readable. It should include more text in the caption of the tables and figures or should just be put into the main manuscript. For example, Fig S4 — cited in line 190. It would be useful to include a sentence after the title of the figure to describe what the reader should take away from that figure.
*We have added more text to the supplementary information and also have updated the captions to make the figures capable of being understood without having to refer to the supplement text. Because of journal limitations, we unfortunately cannot add more figures or tables into the main text of the manuscript.*

**Minor errors**

1. There are inconsistencies in how the manuscript refers to the SI figs (e.g., line 233 vs. line 236) and Figure vs. Fig (for example, see Fig in line 249 and Figure in line 244).
*Thank you for catching these discrepancies. They have been updated.*

2. The font size in every table is way too small
*We have increased the font size for the tables in the main manuscript before forcing a split onto multiple pages.*

3. Extra 5 in line 27 and 14.
*This has been rectified.*

Sincerely,

Shelby M. Scott
National Defense Science and Engineering Graduate Fellow
Department of Ecology and Evolutionary Biology

Louis J. Gross
Chancellor's Professor and Alvin and Sally Beaman Distinguished Professor of Ecology and Evolutionary Biology and Mathematics
Director, National Institute for Mathematical and Biological Synthesis
Director, The Institute for Environmental Modeling, University of Tennessee
Past-President, The Society for Mathematical Biology
